# Changes of diet and dominant intestinal microbes in farmland frogs

**DOI:** 10.1186/s12866-016-0660-4

**Published:** 2016-03-10

**Authors:** Chun-Wen Chang, Bing-Hong Huang, Si-Min Lin, Chia-Lung Huang, Pei-Chun Liao

**Affiliations:** Department of Life Science, National Taiwan Normal University, Taipei, 11677 Taiwan; Taiwan Forestry Research Institute, Technical Service Division, Taipei, 10066 Taiwan

**Keywords:** Abundance, Custom farming, Diet, *Fejervarya limnocharis*, Gut microbiota, Richness

## Abstract

**Background:**

Agricultural activities inevitably result in anthropogenic interference with natural habitats. The diet and the gut microbiota of farmland wildlife can be altered due to the changes in food webs within agricultural ecosystems. In this work, we compared the diet and intestinal microbiota of the frog *Fejervarya limnocharis* in natural and farmland habitats in order to understand how custom farming affects the health of in vivo microbial ecosystems.

**Results:**

The occurrence, abundance, and the numbers of prey categories of stomach content were significantly different between the frogs inhabiting natural and farmland habitats. In addition, differences in the abundance, species richness, and alpha-diversity of intestinal microbial communities were also statistically significant. The microbial composition, and particularly the composition of dominant microbes living in intestines, indicated that the land use practices might be one of factors affecting the gut microbial community composition. Although the first three dominant microbial phyla Bacteroidetes, Firmicutes, and Proteobacteria found in the intestines of frogs were classified as generalists among habitats, the most dominant gut bacterial phylum Bacteroidetes in natural environments was replaced by the microbial phylum Firmicutes in farmland frogs. Increased intestinal microbial richness of the farmland frogs, which is mostly contributed by numerous microbial species of Proteobacteria, Actinobacteria, Acidobacteria, and Planctomycetes, not only reflects the possible shifts in microbial community composition through the alteration of external ecosystem, but also indicates the higher risk of invasion by disease-related microbes.

**Conclusions:**

This study indicates that anthropogenic activities, such as the custom farming, have not only affected the food resources of frogs, but also influenced the health and in vivo microbial ecosystem of wildlife.

**Electronic supplementary material:**

The online version of this article (doi:10.1186/s12866-016-0660-4) contains supplementary material, which is available to authorized users.

## Background

The gastrointestinal tract is the primary site where microorganisms interact with the host species. The gastrointestinal microbiota maintains the functions of nutrient, immune, and development regulation and is important for host health [1–4]. The gut microbiota is commonly influenced by the host diet [3, 5]. The composition of the intestinal microbial community is suggested to result from natural selection operating at the host level to stabilize the gut environment and at the microbial level to promote functional specialization [6]. The relative abundance of symbionts and pathogenic microbes reflects the health status of the host species [7]. Microbial interactions, e.g., resource competition, represent a deterministic factor for the dominance of the gastrointestinal microbial community [8]. Diets serve as a source of gut microbes, which are further selected by the gastrointestinal environment.

The gastrointestinal microbes are composed of autochthonous components (residents) and allochthonous members (hitchhikers from ingested food and waters). Species composition of the gastrointestinal microbial community is different from that of environmental microbes [9]. Firmicutes and Bacteroidetes are the most abundant gastrointestinal microbial phyla [10–14] that were suggested to descend from the early colonists in mammalian gut evolution [6], while Proteobacteria was found to be most dominant in tadpoles [14], house sparrow [15], and fish [16]. The codominance of these phyla was suggested to be a consequence of niche partitioning and metabolic complementation [6]. A healthy gut microbial community occupied by native bacteria could establish a selective environment to prevent emerging pathogens from building up a necessary population size to cause disease. The native microbes can prevent other similar taxa for colonization by high density blocking, and this effect is like the “founder-takes-all” effect of the field of population ecology [17]. However, such “founder-takes-all” effect [17] could be vanished if fast colonization by abundant allochthonous microbes [18]. Here, host immunity not only plays a role as a selective pressure on intestinal microbes but also an object of natural selection by the emerging microbes.

Since the importance of relationships between the food web complexity and species composition in an ecosystem, and between the diet content and intestinal microbiome is emphasized in literature [19], we aimed to explore whether the change in ecosystems as a result of agricultural activities alters the intestinal microbial composition of wildlife. Agricultural activities affect the distribution of wildlife in the natural environment. Farmland biodiversity is typically lower than that in natural fields, which is reflected in a widespread decline of species richness and/or abundance of farmland wildlife [20–23]. Custom farming and agricultural intensification that rely on the use of fertilizers and pesticides recurrently create selective pressures not only on vertebrates, but also on invertebrates [24, 25], plants [26, 27], and soil microbes [28, 29]. The change in land use alters the nutrient cycling of soil, which affects the diversity and abundances of numerous environmentally important genes of microbes [30]. Changes in nutrient cycling affect the food webs of ecosystems [31], and changes in heterotrophic processes and diet diversity can further alter the digestive-tract microbiome of animals [32].

In this study, we used the rice frog *Fejervarya limnocharis* (Gravenhorst, 1829) (Amphibia, Anura, Ranidae) as a system to compare the food composition (diet) and intestinal microbiota between natural and farmland frog populations. *Fejervarya limnocharis* is widespread in East, Southeast, and South Asia. In Taiwan, it is one of the dominant amphibian species in lowland areas and is commonly found in farmlands. The Taiwanese *F. limnocharis* is phylogenetically inferred as a member of the core *F. limnocharis* group based on mtDNA and allozyme evidence [33, 34]. In this study, we profiled the diet and intestinal microbial composition of *F. limnocharis* in natural and farmland habitats. Comparisons of the stomach contents and dominant intestinal microbes were made to understand the changes of intestinal microbiota under the selective pressure of environmental change, such as the habitat variation due to agricultural activities. The multiple comparisons allowed us to address three questions: (1) Is there any dietary difference between frogs living in different habitats? (2) Does the intestinal bacterial diversity differ within and between frogs living in different habitats? (3) Does the compositional change in intestinal microbiota reflect the health of the *in vivo* microbial ecosystem in farmland frogs?

## Results

### Diet differentiation between habitats

A total of 26 *F. limnocharis* individuals (65 %, *n* = 40), of which 17 originated from natural habitats and 9 from farmlands, had stomach contents. A total of 63 individual prey items were identified to 12 orders (Table 1). The prey consumption rate was significantly different between the two sites (*χ*^2^ = 7.03; *P =* 0.008). In the natural habitat, Hymenoptera had the highest index of relative importance (*IRI*) score (1938.05), followed by Orthoptera (459.68). In farmlands, the highest and the second highest *IRI* scores were 2131.69 and 1310.41 in Orthoptera and Coleoptera, respectively (Table 1). The abundance and number of categories of stomach contents were significantly higher in natural habitat than that in farmlands (Z = −2, *P* = 0.045 and Z = −2, *P* = 0.036, respectively; Table 2), but the prey volume was not significantly different between habitats (Z = −1.53, *P* = 0.12; Table 2).

**Table 1:** Stomach contents of *F. limnocharis* in natural habitat and farmlands

	Natural habitat				Farmlands			
Prey category	*N*	%*F*	%*V*	*IRI*	*N*	%*F*	%*V*	*IRI*
Insecta								
Orthoptera	8	6.89	50.39	459.68	4	33.33	35.39	2131.69
Hymenoptera	27	31.03	7.36	1938.05	4	16.66	3.99	542.5
Coleoptera	3	6.89	7.21	91.83	3	25	30.99	1310.41
Blattaria	2	6.89	12.98	117.53	0	0	0	0
Hemiptera	1	3.44	0.09	7.34	0	0	0	0
Lepidoptera (Larvae)	2	6.89	0.9	34.29	0	0	0	0
Dermaptera	0	0	0	0	1	8.33	6.5	113.61
Chilopoda								
Scolopendromorpha	0	0	0	0	1	8.33	14.61	181.24
Malacostraca								
Isopoda	1	3.44	1.65	12.71	0	0	0	0
Arachnida								
Araneae	2	6.89	3.12	49.64	0	0	0	0
Gastropoda								
Stylommatophora	2	6.89	2.66	46.48	1	8.33	8.53	130.51
Oligochaeta	1	3.44	13.64	53.95	0	0	0	0
Total	49				14			

*N* number of prey, *%F* percentage of frequency of each prey item, *%V* percentage of prey volume, *IRI* index of relative importance

**Table 2:** Comparison of stomach contents of rice frog (*Fejervarya limnocharis*) between two habitats by Wilcoxon rank-sum test

		Mean	Std. Dev	Median	Range	Z value	*P*
Number of prey item	Natural habitat	2.82	2.48	2	1 ~ 10	−2	0.045
	Farmlands	1.33	0.71	1	1 ~ 3		
Number of prey category	Natural habitat	1.76	0.9	2	1 ~ 4	−2	0.036
	Farmlands	1.11	0.33	1	1 ~ 2		
Volume of prey(mm^3^)	Natural habitat	134.1	140.04	71.2	2.09 ~ 396.05	−1.53	0.12
	Farmlands	38.77	26.2	32.98	0.78 ~ 78.54		

### Sequencing depth and alpha-diversity of intestinal microbiota

**Fig. 1:**
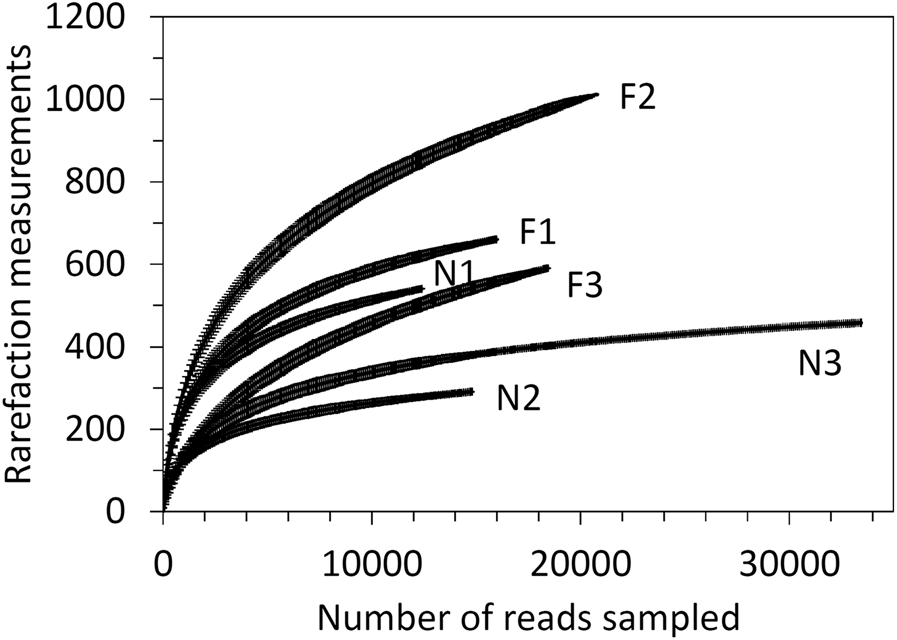
Rarefaction curves for the intestinal microbial communities of rice frogs in natural habitats (N1 ~ N3) and farmland (F1 ~ F3) at a difference level of 3 %

The gut microbes of frog samples from the natural environment (individual labeled with N1 ~ N3) and farmland (individual labeled with F1 ~ F3) were used for exploring the influence of land use practices on intestinal microbial composition. We generated a microbial 16S rRNA dataset consisting of 115,580 filtered high quality, classifiable sequence reads from 133,819 raw sequence reads in total (86.37 %), with a mean number of sequences per frog sample 19,263 ± 6868 (85.42 ± 6.83 %) (Additional file 1: Table S1). The total number of microbial species (OTUs) of intestinal communities characterized using a criterion of >97 % sequence similarity was 1463, with an average length of 496 bps per sequence. The average number of OTUs of each intestinal community was 592 ± 220, ranging from 291 (sample N2) to 1011 (sample F2). The average coverage was 0.733 ± 0.061, ranging from 0.619 (sample F3) to 0.799 (sample N3). The rarefaction analysis indicated that the sequence samplings mostly reached the plateau phase, particularly for farmland frogs (Fig. 1). The microbial community richness and diversity were inferred based on the OTUs characterized using the Abundance Coverage-based Estimator (ACE) and Chao1 indices, which are nonparametric species richness estimator accounting rare species. The ACE of the intestinal microbiota of frogs from natural and farmland populations was 508.696 ± 114.491 and 1006.373 ± 250.663, respectively (significant difference in *t*-test, *t* = 7.503, df = 19, *p* = 4.29e-07, *p* = 2.00e-05 under 99,999 times permutation), whereas the Chao1 index was 522.050 ± 116.238 and 1006.272 ± 268.590, respectively (significant difference in *t*-test, *t* = 3.972, df = 19, *p* = 0.0008, *p* = 0.0009 under 99,999 times permutation). The intestinal microbial community diversities in frogs from both natural and farmland populations were significantly different in terms of Shannon index (585.711 ± 116.074 and 1105.978 ± 289.945, respectively, *t* = 4.559, df = 19, *p* = 0.0002, *p* = 0.0003 under 99,999 times permutation) but not significantly different in Simpson index (3.977 ± 0.660 and 4.438 ± 1.030 respectively, *t* = 0.9947, df = 19, *p* = 0.3324, *p* = 0.3297 under 99,999 times permutation). The detailed estimates of alpha-diversity are shown in Table 3.

**Table 3:** Alpha-diversity of intestinal microbiota of rice frogs (*Fejervarya limnocharis*) at the natural habitat (N1 ~ N3) and farmland (F1 ~ F3)

Sample	OTUs	Coverage	Community richness		Community diversity	
			ACE (95 % CI)	Chao1 (95 % CI)	Shannon index (95 % CI)	Simpson index (95 % CI)
N1	540	0.774	640.451 (609.752, 684.662)	653.554 (611.576, 720.152)	720.152 (4.850, 4.907)	4.907 (0.022, 0.025)
N2	291	0.753	361.315 (334.879, 403.679)	370.875 (334.723, 436.919)	436.919 (3.522, 3.583)	3.583 (0.085, 0.091)
N3	458	0.799	524.321 (501.485, 559.152)	541.720 (507.338, 600.063)	600.063 (3.398, 3.442)	3.442 (0.100, 0.104)
F1	660	0.765	797.248 (759.731, 848.877)	794.101 (749.071, 861.895)	861.895 (4.950, 5.001)	5.001 (0.018, 0.019)
F2	1011	0.687	1358.822 (1287.073, 1449.218)	1385.211 (1289.752, 1513.360)	1513.360 (5.275, 5.320)	5.320 (0.013, 0.014)
F3	590	0.619	863.048 (797.642, 949.057)	839.505 (766.514, 942.678)	942.678 (2.928, 2.993)	2.993 (0.148, 0.155)

*ACE* abundance coverage-based estimator

### Dominant intestinal microbial taxa in rice frogs

**Fig. 2:**
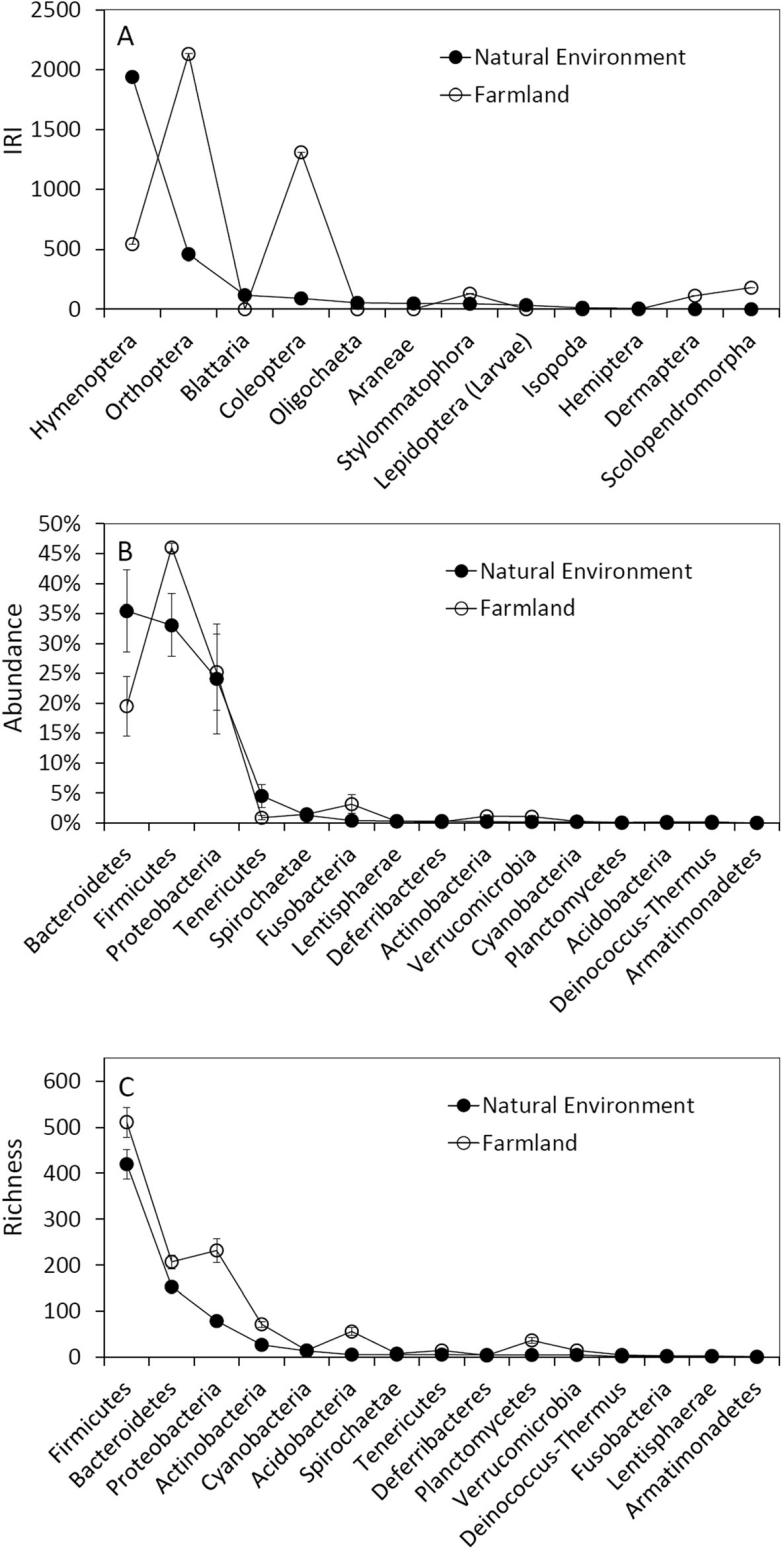
Comparisons of (**a**) the *IRI* of food contents, (**b**) the abundance and (**c**) richness of intestinal microbial phyla between frogs of the natural habitats and farmlands

**Fig. 3:**
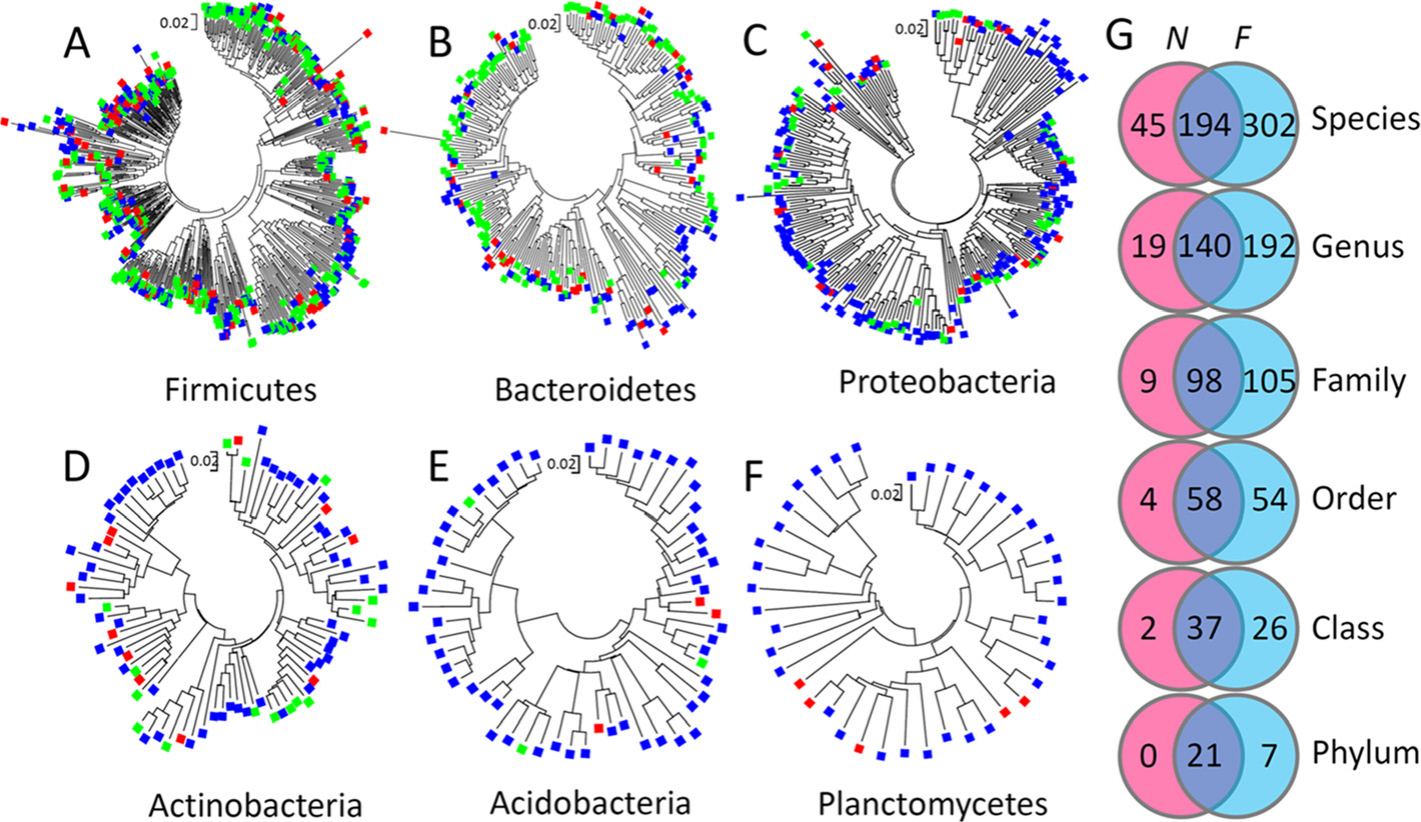
Microbial composition revealed in neighbor-joining trees and Venn diagram. **a–f** The phylogenetic analyses revealed that the microbes of phyla Bacteroidetes and Firmicutes are mostly found in both habitats, while several microbes of phyla Proteobacteria, Acidobacteria, Planctomycetes, and Actinobacteria are only found in farmland frogs. This result suggests that the special intestinal environment leads assemblage of unique microbes with higher phylogenetic niche conservatism in farmland frogs. The red, blue, and green dots indicate the microbes found in frogs of natural population only, farmland population only, and both natural and farmland populations, respectively. **g** Venn diagram representing the species diversity of the intestinal microbial taxa in frog intestines. The numbers in circles represent the number of taxa. As can be seen, a higher number of unique microbial taxa were found in farmland frogs (for which the individual F2 made the highest contribution). These results revealed that the anthropogenic interference have altered the microbial phylogenetic distribution and species composition

**Fig. 4:**
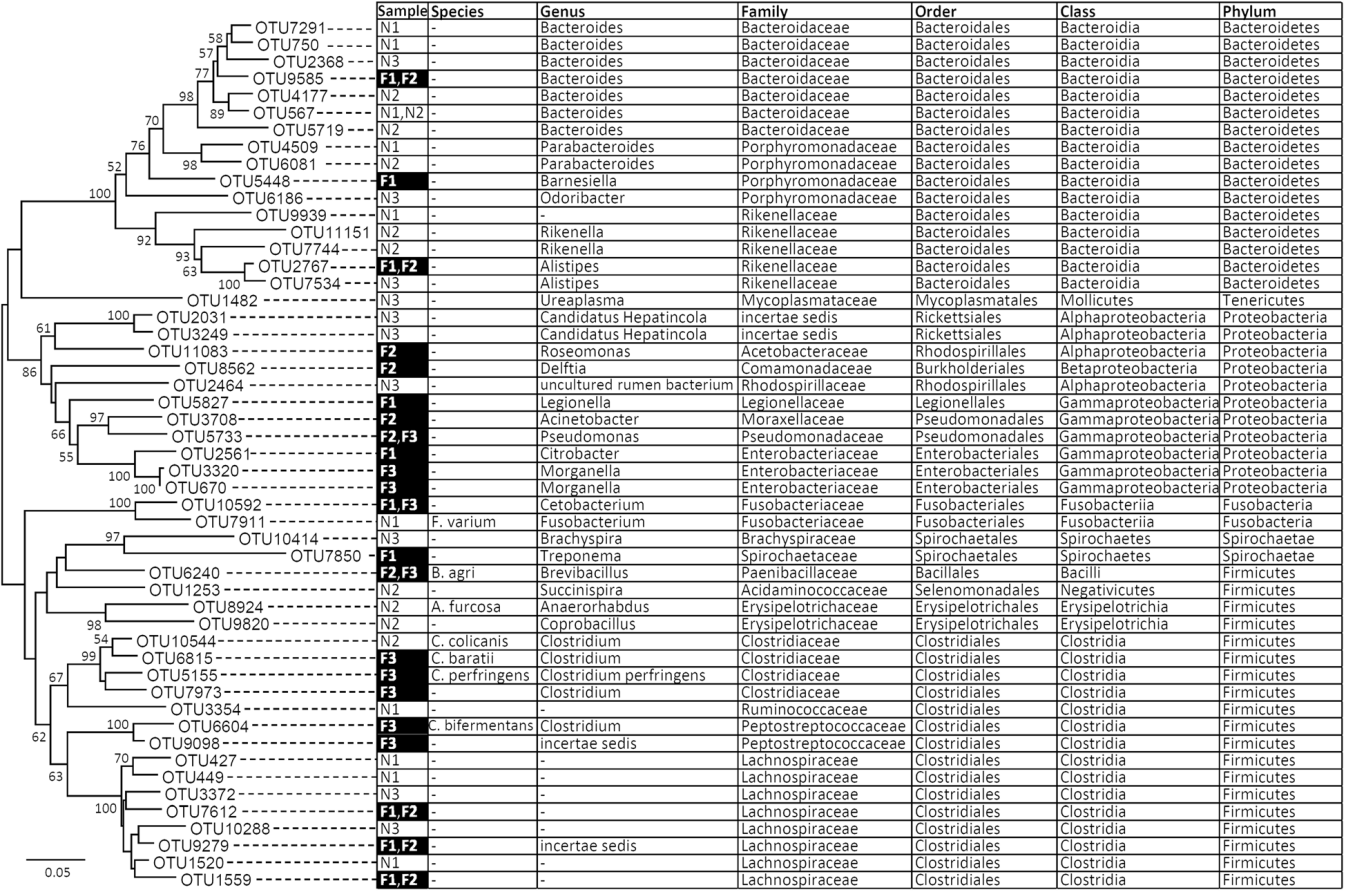
Neighbor-joining analysis for the top 10 species of intestinal microbes of rice frog in natural (N1 ~ N3) and farmland (F1 ~ F3) field ecosystems using the 16S rRNA gene. Taxonomic hierarchies of the identified microorganisms are listed after the NJ tree. The symbol “-” indicates unclassified

The abundance and richness are significantly different between intestinal microbial species richness of *F. limnocharis* from natural and farmland population. The microbes of *F. limnocharis* mainly belonged to the phyla Bacteroidetes, Firmicutes, and Proteobacteria: 35.43, 33.06, and 24.08 % in natural population, and 19.51, 46.02, and 25.22 % in farmland population, respectively (Fig. 2b). Figure 2b showed an obvious increase of abundance in Firmicutes and decrease in Bacteroidetes in farmland frogs compared with those in natural habitats. The three highest-richness phyla of intestinal microbes are Firmicutes, Bacteroidetes, and Proteobacteria (400 ± 60, 153 ± 21, and 79 ± 4 species in natural population; 511 ± 65, 207 ± 28, and 232 ± 51 species in farmland population, respectively, Fig. 2c). Obvious increase richness in Proteobacteria was shown in farmland frogs (Fig. 2c). We compared the identity and differences in microbial taxa between frog samples and between different habitats and found that the farmland frogs were composed of roughly 10 times more unique microbial taxa than the frogs from natural habitat, particularly in the sample F2 (Fig. 3, Additional files 2 and 3: Figure S1 and S2). In addition, there are several farmland-specific clades in Neighbor-Joining (NJ) trees of Proteobacteria, Actinobacteria, Acidobacteria, and Planctomycetes (Fig. 3), indicating that the farmland environments have created special ecological niches for these microbial groups; in contrast, most intestinal microbes of the natural population are commonly found in farmland frogs, indicating that these microbial species retain their ancestral traits. Most of the dominant microbes in frogs from natural habitats were common in farmland frogs. In contrast, many dominant microorganisms in farmland frogs were not found in frogs from natural habitats (Fig. 3, Additional file 1: Table S2). This comparison suggests a replacement of dominant intestinal microbes in farmland frogs. The phylogenetic analysis also showed consistent results that the main composition of intestinal microbes of the rice frogs was different between the natural and farmland habitats. The phylogenetic grouping of the top 10 microbes indicated an admixture of dominant microbes collected from the same habitat, but represented apparent sorting of microbes in different habitats. Taxa in the phyla Bacteroidetes and Firmicutes dominated the guts of frogs from the natural population (N1 ~ N3), whereas taxa from the phyla Proteobacteria and Firmicutes dominated the intestines of the farmland frogs (Fig. 4).

### Classification of gut bacteria

Intestinal microbial organisms were classified into three categories—generalists, specialists, and too rare—based on the multinomial species classification method. Eight phyla were habitat generalists (Firmicutes, Bacteroidetes, Proteobacteria, Spirochaetae, Lentisphaerae, Deferribacteres, Cyanobacteria, and Planctomycetes), ten were farmland specialists (Fusobacteria, Actinobacteria, Verrucomicrobia, Deinococcus-Thermus, Acidobacteria, Elusimicrobia, Synergistetes, Chloroflexi, Gemmatimonadetes, and one unclassified phylum), one was a natural-habitat specialist (Tenericutes), and ten phyla were too rare to be identified (Additional file 1: Table S3 and Additional file 4: Figure S3). At the species level, we found that 44 (8.1 %), 34 (6.3 %), 82 (15.2 %), and 381 (70.4 %) OTUs belonged to the generalist, natural-habitat specialist, farmland specialist, and too-rare types, respectively (Additional file 1: Table S4 and Additional file 4: Figure S3). This classification indicated that relatively low proportions of common gastrointestinal bacteria are found in both natural and farmland habitats and that approximately one-fifth of the bacteria differentiate the gut microbiota between habitats, while most microorganisms are rare in these habitats. We also performed pairwise comparisons of the gut microbial community of different host individuals and found a relatively high proportion of generalists and a lower proportion of specialists in gut bacterial communities of frogs within habitats (generalists: 13.97 % ± 1.59 %; specialists: 26.62 % ± 2.98 %) than those seen for frogs between habitats (generalists: 9.67 % ± 0.99 %; specialists: 30.83 % ± 1.28 %). However, these differences were not statistically significant, indicating that the individual effect cannot be neglected while explaining gut microbial diversity (Additional file 5: Figure S4).

## Discussion

The differences in stomach contents of rice frogs may reflect the changes in faunal species composition. There were long-term anthropogenic disturbance such as the use of fertilizers and pesticides in custom farming. These anthropogenic activities may alter the faunal species composition in farming habitat although the invertebrate fauna of both natural and farmlands were not directly investigated. The diet analysis showed that the food volume of rice frogs was not significantly different between the natural and farmland habitats, but the stomach content (abundance and number of prey categories) in farmland frogs showed lower diversity than that in frogs from natural habitats. Such dietary alteration is probably ascribed to agricultural activities. The increase of soil life (700 % increase of megafauna, 2500 % increase of nematodes, and 70 % increase of soil microorganisms) in 14-year of monitoring conservation agriculture and organic farming indicated the harmful effects of pesticides and fertilizers on the terrestrial ecosystem [35]. In this study, the decreased number of prey categories in the guts of farmland frogs did not only point to the presence of simplified invertebrate and plant communities in farmlands and the decreased *IRI* of Hymenoptera (Fig. 2a), but also reflected the reduction of pollinator demand in the plant community [36]. In contrast, the increase in the *IRI* of Orthoptera (Fig. 2a) often suggested an increase in monocot abundance, particularly of the Poaceae [37–39]. The dietary analysis suggested the occurrence of dramatic changes in the farmland ecosystem due to agricultural activities.

Conventional agriculture or custom farming may strongly alter the species composition in many ways [35]. For example, habitat destruction during farming may reduce diet resources and subsequently alter gastrointestinal microbiome composition in the howler monkey (*Alouatta pigra*) [40]. Habitat degradation following temperature change [41] and water pollution [42] can also subsequently alter amphibian gut microbiota. Research on the microbial composition surrounding the forest soils revealed similar dominant microbes among different forest types with slight difference in microbiota [43]. This is similar to our findings that the dominant gut bacteria are habitat generalists and most gut microbes belong to the “too-rare” type bacteria. However, we found that approximately one-fifth of the gut bacteria were habitat specialists (Additional file 1: Table S3 and S4), indicating that the varied habitat indeed altered the gut microbial composition.

Our results suggest that the estimated intestinal microbial species richness in frog guts varied within the same order of magnitude as that in the human gut, as estimated based on fecal analysis [10]. The gut microbiota is mainly determined by the environmental conditions where the host species reside [44]. Although relatively few frog samples were chosen to represent the gut microbiota of rice frogs, the gut environments of different frog individuals from the same habitat may be similar because frogs from the same habitat might share similar niches. The digestive fluid secreted from some specialized cells in the epithelium of the small intestine, which maintains a stable environment in the gut [45, 46]. Therefore, the dominant gut bacteria could be similar among frogs with similar habitats because the coexistence of these dominant bacteria reflects the consequence of long-term selection in the gut environment. We also found that both dominant gut bacterial species in the natural habitat (Candidatus Hepatincola, uncultured α-Proteobacterium, total of 21.02 %) and farmland habitat (*Morganella* sp., total of 10.95 %) were habitat specialists, indicating that the external environment plays a major role in the gut-dominant bacteria in frogs.

On comparing the microbial species composition, a certain proportion of common microbial taxa (approximately 1/3 ~ 1/2) was similar between frogs from natural habitats and farmlands, revealing the host species specificity of microbes (Additional file 3: Figure S2). The same pattern of similar taxa in different microbial communities of diet-differentiated, geographically distant host populations was also found in wild and laboratory *Drosophila* [47] and *Cylindroiulus fulviceps* (Diplopoda) with different feeding treatments [48]. However, the higher proportion of bacterial habitat specialists than habitat generalists suggested that external factors such as diet, geographic variation, and the environment still play major roles in determining the gut microbial composition (Additional file 4: Figure S3). Differences in dominant taxa, revealed by different clusters in phylogenetic analysis (Fig. 4), suggested strong selectivity of the microbial community in the gut environment [49].

The microbial abundances between frogs of natural habitat and farmland are different, especially in Bacteroidetes and Firmicutes. These two phyla are the most prevalent bacteria in digestive tracts of terrestrial animals [50, 51]. In natural population, Bacteroidetes are the mostly abundant microbes in rice frogs’ intestines followed by Firmicutes, but the abundance of Bacteroidetes is lower than Firmicutes in farmland population (Fig. 2b). The Bacteroidetes of the frogs of natural habitats were mostly composed of the order Bacteroidales (Fig. 4). Bacteroidales are known as symbiotic bacteria essential for the digestive activity of several organisms [1, 16, 25, 48, 52, 53]. However, the abundance of Bacteroidetes decreased in farmland frogs and was replaced by Firmicutes (Fig. 2b). Higher Firmicutes-to-Bacteroidetes ratio could improve the efficiency of calories uptake from food [54]. These Firmicutes microbes were almost composed of Ruminococcaceae and Lachnospiraceae, which digest cellulose and ferment glucose and xylose to obtain nutrients and are the prevalent bacterial families in herbivore’s digestive system [55]. The ecological meaning of the increased abundance of Ruminococcaceae and Lachnospiraceae in farmland frogs’ intestine is unknown yet, but the high Firmicutes-to-Bacteroidetes ratio suggests that the alteration of food composition might change the intestinal environments and microbial community in farmland frogs.

The differences in dominant intestinal microbial taxa between natural habitats and farmlands revealed in NJ analysis indicated that the frogs acquired different bacteria due to the changes in environmental conditions. In contrast to the high frequency of common microbial taxa in frogs from natural habitats, the intestinal microbial communities of farmland frogs were characterized by higher ratios of unique microbes (Additional files 2 and 3: Figure S1 and S2). These unique microbes mostly belonged to Proteobacteria, Actinobacteria, Acidobacteria, and Planctomycetes (Fig. 3). From the analysis of the top 10 microbial organisms of every frog intestinal samples, we found that the Classes of γ-Proteobacteria in farmland frogs have an obvious increase in abundance (19.75 %) than the frogs of natural habitats (0.05 %). Although γ-Proteobacteria is common in guts of diverse taxa including amphibians [e.g., House Sparrow (*Passer domesticus*) [15], leopard frog (*Lithobates pipiens*) [14]], most of the γ-Proteobacteria in farmland frogs belong to the order Enterobacteriales (72.91 %) (Fig. 4). Several intestinal bacteria that are dominant in the farmland frogs, such as species in the genera *Treponema* (Spirochaetes), *Roseomonas* (α-Proteobacteria), *Clostridium* (Firmicutes) and genera *Legionella*, *Acinetobacter*, *Pseudomonas, Citrobacter*, *Morganella* of γ-Proteobacteria (Fig. 4), were probably infectious disease-related pathogens. These bacterial genera are clinically proven to cause emerging infectious diseases (EID) not only in humans (e.g., *Legionella* [56, 57]; *Acinetobacter* [57–59]; *Pseudomonas* [60, 61]; *Citrobacter* [62, 63]; *Morganella* [64, 65]; *Treponema* [66, 67]; *Roseomonas* [68–70], and *Clostridium* [71, 72]), but also in amphibian (for example, *Acinetobacter* would cause ulceration and necrosis in *Rhinoderma darwini* [73], *Pseudomonas* and *Citrobacter* might induce immune-response in *Rana pipiens* [74, 75], and so did the *Morganella* in *Andrias davidianus* [76].)

These bacteria mostly belong to the Proteobacteria. Several Proteobacteria have pathogenic or antipathogenic functions in amphibians, for example, *Janthinobacterium lividum* in amphibian guts can inhibit the growth of lethal amphibian fungi [77, 78]. Certain antifungal bacteria from the genus *Pseudomonas* that were discovered on the skin of amphibians, that is, the salamander (*Plethodon cinereus* and *Hemidactylium scutatum*) [79, 80] and the frog (*Rana muscosa*) [81]), were also found in leopard frogs (*Rana pipiens*) [82]). The intestines of amphibians could serve as the reservoir for these antifungal bacteria via uptake of invertebrates that have come into contact with these bacteria in the soil or by eating their own shed skin [77]. The dynamics of these bacteria may be an indicator of host susceptibility to these lethal fungi [83]. These Proteobacteria were previously found in smaller amounts than Bacteroidetes and Firmicutes in healthy adult mammals [19] and human gastrointestinal samples [6] and in the frogs from natural habitats investigated in this study.

In addition to the phylum Proteobacteria, certain bacterial genera, such as *Flavobacterium* in phylum Bacteroidetes and *Actinobacterium* and *Bacillus* in phylum Firmicutes, that are chytrid-resistant or have probiotic capabilities were mostly found in the gut of frogs in the farmland habitat (sequence reads = 21, 59, and 4 in *Flavobacterium*, *Actinobacterium*, and *Bacillus*, respectively) but rarely in the natural habitat (sequence reads = 1, 4, and 0, respectively). The higher proportion of pathogen-resistant bacteria found in farmland frogs reflected the higher occurrence rate of harmful pathogens in the farmland habitat. The intestinal microbial composition in frogs from farmlands was possibly under a high risk of infectious diseases. Because stomach content is a proxy of food source in the natural environment, the differences in intestinal microbiota between farmland and natural habitats did not only explain the dietary alteration, but also reflected the risk of disease in farmland wildlife due to the ecosystem alterations as a result of anthropogenic activities.

Intestinal microbiota can possibly reflect the state of the immune system and health of the host species [2]. Different microbial composition in frog guts may be related to pathogen resistance, for example, differential antifungal bacterium composition between populations may be important in preventing chytrid fungus infections [84]. Differentiation of gut microbial composition could even reflect overall body healthy of frogs. We found lower food diversity but higher intestinal microbial richness in farmland frogs than in natural habitats. This can possibly be explained by the recurrent selective pressures from agricultural activities simplifying the farmland fauna and increasing the risks of infectious disease in frog predators. Simplified food sources could also weaken the immunity of wild animals and lead to higher pathogen invasion [85]. Disturbance in gut microbial composition and microbial and ecological dynamics can lead to an increased risk of disease outbreaks in wildlife [86], or provide less anti-pathogen reservoir for preventing infection of skin pathogen [84]. Anthropogenic ecosystem alteration and pathogen or vector movements via human or natural agencies could give rise to EID in wildlife [87, 88]. In this study, we showed that anthropogenic interference in ecosystem (such as agricultural activities) and a change of “vectors” (food contents) can weaken frog immunity, which is reflected in the change of intestinal microbial climax.

## Conclusions

Alterations in diet and intestinal microbial composition in farmland *F. limnocharis* indicate that custom farming influences the intestinal ecosystem of wildlife. Food may not only play a resource role but also be one of the factors serve as a vector of infectious microbes. The intestinal microbial composition reflected the result of intestinal environmental selection by both extrinsic (environment, such as habitat disturbance, temperature, and food) and intrinsic (immune system) factors. In the farmland habitat, less diverse food content and more diverse intestinal microbiota in frogs were found than in natural habitats. This result suggests that the in vitro ecosystem changes in vivo ecosystem. The intestinal microbiota of *F. limnocharis* was determined by both environmental factors and host species, whereas the dominant intestinal microbes were more easily affected by external environmental conditions than were the rare microbes. The increased numbers of Proteobacteria suggested that pathogenic invasion was affected or will be affected by the weakened immunity of farmland frogs, which is probably caused by the heavy use of pesticides and fertilizers in farmland. The current study revealed the change in food resources and intestinal microbial diversity in farmland wildlife, and also suggested that outbreaks of disease-related bacteria within the gut microbial community can reflect the damage of in vivo and in vitro ecosystem health due to agricultural interference. It should be notified that the small sample size of frogs in this study may not be sufficient to draw strong conclusions, but could be indicative of changes across habitats. Even though, this research still represents different microbial composition between habitats and provides a reference for future studies regarding the amphibians’ gastrointestinal microbiota.

## Methods

### Sampling system

*Fejervarya limnocharis* is a medium-sized frog with an average body length of 4.3 cm and 5.5 cm in males and females, respectively [89]. This species is widespread in lowland areas at altitudes below 1500 m in Taiwan. *F. limnocharis* generally aggregates around breeding ponds during the breeding season from spring to summer. *F. limnocharis* is tolerant to human disturbance, and therefore is commonly found in farmlands. Forty frog samples were collected from Hualin experimental forest (the natural habitat, 24°53′N, 121°33′E) and farmlands in Quchi Community (24°55′N, 121°32′E) in Xindian (Dist., New Taipei City, Taiwan) on September 27^th^, 2012. The distance between these two sites is less than 3 km. All frog samples were brought to the laboratory of Nation Taiwan Normal University for immediate profiling of diets and intestinal microbial composition.

### Diet analyses

Frog samples were sacrificed and preserved in 70 % ethanol. Stomach content of frogs (prey) was collected via dissection and stored in 70 % ethanol. The length and width of the stomach content items were measured using cartesian papers and the prey items were identified to order level under a stereomicroscope. The volume of prey items was estimated using the formula proposed by Dunham [90]. For assessing the importance of each consumed prey category, the *IRI* was calculated based on the formula *IRI* = %*O* (%*N* + %*V*) [91], where %*O*, %*N*, and %*V* are the percentages of the occurrence, relative abundance, and measured volume of each prey category, respectively, in all stomachs. The intake rate (which describes the prey eaten by *F. limnocharis*) between habitats was compared using the Chi-square test. The numbers, categories, and volumes of stomach contents were compared between habitats using Wilcoxon rank-sum test. All statistical analyses were conducted using JMP 7.0.

### Intestinal microbiota

The gut microbes of six frog samples from the natural environment (N1 ~ N3) and farmland (F1 ~ F3) were used for exploring the influence of food composition on intestinal microbial composition. Intestinal microbial metagenomic DNA was extracted based on a protocol described by Sharma et al. [92]. For each sample, we amplified the V4 hypervariable 16S rRNA region using the primer set 27 F (5′-AGAGTTTGATCCTGGCTCAG-3′) and 533R (5′-TTACCGCGGCTGCTGGCAC-3′). The DNA library was constructed according to the Roche GS FLX Titanium emPCR kit (Roche Applied Science). Pyrosequencing was carried out by Welgene Biotech Co., Ltd. (Taipei, Taiwan) using a Roche 454 FLX titanium instrument and reagents following the manufacturer’s instructions. The V4 sequence fragments shorter than 200 bp, without barcodes, with polyN or polyA/T, and the reads with < Q25 were removed. Sequences with >97 % identity were treated as the same species and as an operational taxonomic unit (OTU). Each OTU was classified by annotating to the SILVA database. The alpha-diversity of the gut microbiota was estimated using community richness indices, i.e., the ACE [93], the Chao1 index [50, 94], and community diversity indices, i.e., the Shannon index and Simpson index. Indices ACE, Chao1, Shannon index and Simpson index between habitats were compared. Because the small sample size could result in non-normal distribution, we regenerated 20 normalized pseudodata based on the mean and standard deviation from the observed data, and performed 99,999 times permutations for a one-sample randomization test on differences of values between habitats. Validation of *t*-test was given by similar *p*-values before and after permutations. Rarefaction analysis was executed to measure how the gut microbial composition of the rice frog varied depending on the sample size.

After determination of the microbial diversity, the differences in the gut microbial composition of rice frogs between the natural and the farmland populations was examined by phylogenetic analysis. The neighbor-joining (NJ) method was used for constructing a genetic-distance tree to elucidate the genetic distribution patterns of intestinal microbes between different habitats. Nucleotide sequence alignments were performed using the Clustal W program. The evolutionary distances were computed using the *p*-distance method. The NJ tree was constructed using the program MEGA v. 5.05 [51]. The NJ relationships of six dominant microbial phyla Bacteroidetes, Firmicutes, Proteobacteria, Planctomycetes, Actinobacteria, and Acidobacteria were reconstructed separately for revealing the grouping patterns of intestinal microbes between different habitats. In addition, every top 10 microbial OTUs of frogs (yielding a total 51 OTUs) were used to construct a genetic-distance tree using the NJ method for clearly representing the systematic positions of the dominant intestinal microbes of frogs in natural habitats and farmlands, with the following settings: the composite likelihood substitution model [95], the uniform rate among sites, the heterogeneous rates among lineages and complete deletion of gaps. A 1000 bootstrap replication was used to evaluate the supporting values for lineage grouping.

We also used the multinomial species classification method (CLAM) [96] to classify the bacteria of generalists and specialists in two distinct habitats with the vegan package in R. CLAM is a kind of two-group species classification method. The supermajority rule that uses the specialization threshold value 2/3 was adopted for determining the bacteria of habitat specialists. Under the supermajority rule, the minimum abundance for classification (i.e., coverage limit) was estimated and the taxa that had abundance below the coverage limit were considered as “too rare”. CLAM was also used for pairwise comparison between host individuals within habitats and between habitats. Comparisons of the proportions of generalists and specialists between “within habitats” and “between habitats” aid in understanding the degree of individual effect on gut microbial diversity.

### Availability of data and materials

The 16S rDNA sequences identified in this study have been deposited in the NCBI GenBank under the Bioproject PRJNA279212 (Accession number: SRX965751). Frog samples were stored in the specimen room of Department of Life Science, National Taiwan Normal University.

### Ethics statements

Protocols of this study and the animal use were reviewed and approved by the Ethics Committee of National Pingtung University of Science and Technology (NPUST) (Approved No. NPUST-101-079) and the Institutional Animal Care and Use Committee of National Taiwan Normal University (Approved No. 101024).

## Additional files

Additional file 1:
**Table S1.** Summary of sequence reads used in this study. **Table S2.** Percentages of top 10 microbial taxa of every rice frog (*Fejervarya limnocharis*) sample. **Table S3.** List of categories of the habitat generalists, specialists and too-rare types at the phylum level. **Table S4.** List of categories of the habitat generalists, specialists and too-rare types at the species level. (DOCX 129 kb) Additional file 2: Figure S1. Venn diagram representing the species diversity of the intestinal microbial taxa in frog intestines. The numbers in circles represent the number of taxa. As can be seen, a higher number of unique microbial taxa were found in farmland frogs (for which the individual F2 made the highest contribution). (TIF 17137 kb) Additional file 3: Figure S2. The Neighbor-joining tree reveals the phylogenetic position of the intestinal microbes of *Fejervarya limnocharis* in farmland only (blue), in natural habitats only (red), and in both environments (green). The phylogenetic analysis showed that roughly 1/3 clades of microbial species are found in the farmland frogs only. (TIF 15121 kb) Additional file 4: Figure S3. Categories of gut bacteria between habitats classified by multinomial species classification method (CLAM). Classification in phylum level and species level are shown. Values in plots indicate the number of bacterial taxa (i.e. phyla and species) of each category. F and N in the x- and y-axes indicate the farmland and natural habitat, respectively. (TIF 156 kb) Additional file 5: Figure S4. Pairwise comparison of gut bacterial categories of individual hosts within habitats and between habitats. All three comparisons of generalists, specialists, and too-rare type of bacterial communities within habitats and between habitats are non-significant different. (TIF 3668 kb)
