# Supplementary material for: Changes of diet and dominant intestinal microbes in farmland frogs
Source: BMC Microbiol. 2016 Mar 10;16:33. doi: 10.1186/s12866-016-0660-4 (PMC4785643; doi:10.1186/s12866-016-0660-4)
Supplement: Additional file 1: — Table S1. Summary of sequence reads used in this study. Table S2. Percentages of top 10 microbial taxa of every rice frog (Fejervarya limnocharis) sample. Table S3. List of categories of the habitat generalists, specialists and too-rare types at the phylum level. Table S4. List of categories of the habitat generalists, specialists and too-rare types at the species level. (DOCX 129 kb) [file 12866_2016_660_MOESM1_ESM.docx]

**Changes of diet and dominant intestinal microbes in the rice frog *Fejervarya limnocharis* (Amphibia, Anura, Ranidae) in farmland**

Chun-Wen Chang^a,b,†^, Bing-Hong Huang^a,†^, Si-Min Lin^a^, Chia-Lung Huang^a^, Pei-Chun Liao^a,*^

1. Department of Life Science, National Taiwan Normal University, Taipei 11677, Taiwan
2. Taiwan Forestry Research Institute, Technical Service Division, Taipei 10066, Taiwan

**Additional file 1: Supplementary Materials**

**Table S1** Summary of sequence reads used in this study.

**Table S2** Percentages of top10 microbial taxa of every rice frog (*Fejervarya limnocharis*) sample.

**Table S3** List of categories of the habitat generalists, specialists and too-rare types at the phylum level.

**Table S4** List of categories of the habitat generalists, specialists and too-rare types at the species level.

**Table S1** Summary of sequence reads used in this study.

|  | Total reads | After 1st cleaning^a^ | After 2nd cleaning^b^ | % reads after cleaning | OTUs |
| --- | --- | --- | --- | --- | --- |
| N1 | 17442 | 13700 | 12380 | 70.98% | 540 |
| N2 | 17343 | 15656 | 14734 | 84.96% | 291 |
| N3 | 37167 | 34860 | 33441 | 89.97% | 458 |
| F1 | 18438 | 16714 | 15906 | 86.27% | 660 |
| F2 | 23334 | 21971 | 20716 | 88.78% | 1011 |
| F3 | 20095 | 18963 | 18403 | 91.58% | 590 |
| Sum | 133819 | 121864 | 115580 | 86.37% | 3550 |

^a^ Removing the sequence reads without barcodes, cannot be identified, and the reads with length < 70bp.

^b^ Removing the sequence reads with adaptors in 3’ends, with polyN and poltA/T. The reads with sequence length < 200bp and the base with quality score <Q25 were discarded.

**Table S2** Percentages of top10 microbial taxa of every rice frog (*Fejervarya limnocharis*) sample.

| Taxon | N1 | Taxon | N2 | Taxon | N3 | Taxon | F1 | Taxon | F2 | Taxon | F3 |
| --- | --- | --- | --- | --- | --- | --- | --- | --- | --- | --- | --- |
| **Species level** |  |  |  |  |  |  |  |  |  |  |  |
| **top10** | **71.71%** |  | **81.17%** |  | **84.27%** |  | **62.03%** |  | **57.72%** |  | **84.91%** |
| **top5** | **60.87%** |  | **64.68%** |  | **73.40%** |  | **45.16%** |  | **42.49%** |  | **73.25%** |
| Lachnospiraceae unclassified | 17.93% | Parabacteroides unclassified | 26.45% | Candidatus Hepatincola uncultured alpha proteobacterium | 37.70% | Lachnospiraceae unclassified | 11.46% | Lachnospiraceae unclassified | 12.09% | Morganella unclassified | 32.22% |
| Parabacteroides unclassified | 16.77% | Bacteroides unclassified | 16.91% | Lachnospiraceae unclassified | 15.03% | Ruminococcaceae unclassified | 10.33% | Bacteroides unclassified | 10.90% | Peptostreptococcaceae unclassified | 15.00% |
| Ruminococcaceae unclassified | 11.79% | Clostridium colicanis | 9.32% | Bacteroides unclassified | 9.14% | Bacteroides unclassified | 9.49% | Ruminococcaceae unclassified | 8.65% | Clostridium perfringens | 14.29% |
| Bacteroides unclassified | 10.79% | Coprobacillus uncultured bacterium | 6.91% | Ureaplasma unclassified | 8.05% | Cetobacterium uncultured bacterium | 7.76% | Brevibacillus agri | 6.36% | Morganella uncultured morganella sp. | 6.74% |
| Lachnospiraceae uncultured bacterium | 3.59% | Ruminococcaceae unclassified | 5.09% | Ruminococcaceae unclassified | 3.48% | Legionella sp. | 6.12% | Lachnospiraceae uncultured bacterium | 4.49% | Clostridium uncultured bacterium | 5.00% |
| Rikenellaceae unclassified | 2.42% | Lachnospiraceae unclassified | 4.83% | Alistipes unclassified | 3.40% | Lachnospiraceae uncultured bacterium | 4.92% | Parabacteroides unclassified | 3.87% | Clostridium baratii | 4.12% |
| Bacteroides uncultured bacterium | 2.35% | Rikenella uncultured bacterium | 3.60% | Rhodospirillaceae uncultured rumen bacterium | 2.13% | Parabacteroides unclassified | 3.75% | Pseudomonas unclassified | 3.35% | Cetobacterium uncultured bacterium | 2.47% |
| Rikenella uncultured bacterium | 2.15% | Rikenella unclassified | 3.41% | Brachyspira unclassified | 1.98% | Alistipes uncultured bacterium | 3.17% | Alistipes uncultured bacterium | 2.80% | Bacteroides unclassified | 2.04% |
| Parabacteroides uncultured bacterium | 2.05% | Bacteroides uncultured bacterium | 2.37% | Odoribacter unclassified | 1.74% | Citrobacter unclassified | 2.94% | Anaerotruncus unclassified | 2.63% | Lachnospiraceae unclassified | 1.91% |
| Fusobacterium varium | 1.87% | Alistipes unclassified | 2.28% | Rikenellaceae unclassified | 1.62% | Erysipelotrichaceae unclassified | 2.09% | Roseomonas unclassified | 2.58% | Ruminococcaceae unclassified | 1.12% |
| **Genus level** |  |  |  |  |  |  |  |  |  |  |  |
| **top10** | **76.80%** |  | **85.76%** |  | **83.43%** |  | **60.09%** |  | **56.06%** |  | **86.66%** |
| **top5** | **57.72%** |  | **71.34%** |  | **70.90%** |  | **37.38%** |  | **35.35%** |  | **80.30%** |
| Parabacteroides | 18.88% | Parabacteroides | 26.68% | Candidatus Hepatincola | 37.70% | Bacteroides | 9.74% | Bacteroides | 11.14% | Morganella | 38.96% |
| Bacteroides | 13.17% | Bacteroides | 19.40% | Bacteroides | 10.50% | Cetobacterium | 7.76% | Lachnospiraceae uncultured | 6.47% | Lachnospiraceae incertae sedis | 14.99% |
| Lachnospiraceae unclassified | 11.77% | Clostridium | 11.32% | Lachnospiraceae uncultured | 8.62% | Ruminococcaceae unclassified | 7.12% | Brevibacillus | 6.36% | Peptostreptococcaceae incertae sedis | 14.57% |
| Lachnospiraceae uncultured | 7.20% | Rikenella | 7.03% | Ureaplasma | 8.05% | Lachnospiraceae uncultured | 6.60% | Lachnospiraceae incertae sedis | 5.80% | Clostridium | 9.31% |
| Ruminococcaceae unclassified | 6.70% | Coprobacillus | 6.91% | Lachnospiraceae unclassified | 6.03% | Legionella | 6.16% | Ruminococcaceae unclassified | 5.58% | Cetobacterium | 2.47% |
| Lachnospiraceae incertae sedis | 4.71% | Lachnospiraceae unclassified | 3.83% | Alistipes | 3.67% | Lachnospiraceae incertae sedis | 5.48% | Lachnospiraceae unclassified | 4.96% | Bacteroides | 2.11% |
| Anaerotruncus | 4.36% | Alistipes | 2.96% | Odoribacter | 2.51% | Alistipes | 4.96% | Alistipes | 4.77% | Peptostreptococcaceae unclassified | 1.26% |
| Rikenella | 3.78% | Ruminococcaceae unclassified | 2.91% | Ruminococcaceae unclassified | 2.24% | Lachnospiraceae unclassified | 4.78% | Parabacteroides | 4.19% | Parabacteroides | 1.05% |
| Ruminococcaceae incertae sedis | 3.13% | Erysipelotrichaceae uncultured | 2.48% | Rhodospirillaceae uncultured | 2.13% | Parabacteroides | 4.00% | Acinetobacter | 3.43% | Lachnospiraceae unclassified | 0.98% |
| Ruminococcaceae uncultured | 3.10% | Succinispira | 2.24% | Brachyspira | 1.98% | Ruminococcaceae incertae sedis | 3.49% | Pseudomonas | 3.36% | Acinetobacter | 0.96% |
| **Family level** |  |  |  |  |  |  |  |  |  |  |  |
| **top10** | **91.91%** |  | **96.97%** |  | **96.02%** |  | **83.26%** |  | **78.52%** |  | **91.72%** |
| **top5** | **84.48%** |  | **79.03%** |  | **78.81%** |  | **60.04%** |  | **58.38%** |  | **83.60%** |
| Lachnospiraceae | 23.81% | Porphyromonadaceae | 27.01% | Rickettsiales incertae sedis | 37.95% | Lachnospiraceae | 16.86% | Lachnospiraceae | 17.28% | Enterobacteriaceae | 39.13% |
| Porphyromonadaceae | 21.28% | Bacteroidaceae | 19.40% | Lachnospiraceae | 15.38% | Ruminococcaceae | 16.75% | Ruminococcaceae | 15.37% | Lachnospiraceae | 16.86% |
| Ruminococcaceae | 17.90% | Clostridiaceae | 11.32% | Bacteroidaceae | 10.50% | Bacteroidaceae | 9.74% | Bacteroidaceae | 11.14% | Peptostreptococcaceae | 15.83% |
| Bacteroidaceae | 13.17% | Erysipelotrichaceae | 10.86% | Mycoplasmataceae | 8.05% | Porphyromonadaceae | 8.93% | Porphyromonadaceae | 7.64% | Clostridiaceae | 9.31% |
| Rikenellaceae | 8.32% | Rikenellaceae | 10.44% | Rikenellaceae | 6.93% | Fusobacteriaceae | 7.76% | Paenibacillaceae | 6.95% | Fusobacteriaceae | 2.47% |
| Fusobacteriaceae | 1.87% | Ruminococcaceae | 6.20% | Ruminococcaceae | 5.44% | Rikenellaceae | 6.44% | Rikenellaceae | 6.48% | Bacteroidaceae | 2.11% |
| Erysipelotrichaceae | 1.70% | Lachnospiraceae | 5.81% | Porphyromonadaceae | 5.16% | Legionellaceae | 6.16% | Erysipelotrichaceae | 3.92% | Ruminococcaceae | 2.03% |
| Veillonellaceae | 1.49% | Acidaminococcaceae | 2.24% | Rhodospirillaceae | 3.42% | Erysipelotrichaceae | 4.28% | Moraxellaceae | 3.62% | Porphyromonadaceae | 1.97% |
| Clostridiales xiii incertae sedis | 1.30% | Desulfovibrionaceae | 2.12% | Brachyspiraceae | 1.98% | Enterobacteriaceae | 4.17% | Pseudomonadaceae | 3.36% | Moraxellaceae | 1.02% |
| Desulfovibrionaceae | 1.07% | Veillonellaceae | 1.57% | Veillonellaceae | 1.21% | Desulfovibrionaceae | 2.17% | Comamonadaceae | 2.76% | Rikenellaceae | 0.99% |
| **Order level** |  |  |  |  |  |  |  |  |  |  |  |
| **top10** | **97.54%** |  | **99.52%** |  | **98.92%** |  | **91.94%** |  | **91.34%** |  | **96.54%** |
| **top3** | **89.25%** |  | **92.13%** |  | **82.66%** |  | **69.64%** |  | **68.60%** |  | **88.63%** |
| Clostridiales | 44.56% | Bacteroidales | 56.88% | Rickettsiales | 37.95% | Clostridiales | 36.18% | Clostridiales | 35.69% | Clostridiales | 44.36% |
| Bacteroidales | 42.77% | Clostridiales | 24.39% | Bacteroidales | 23.21% | Bacteroidales | 25.68% | Bacteroidales | 25.75% | Enterobacteriales | 39.13% |
| Selenomonadales | 1.92% | Erysipelotrichales | 10.86% | Clostridiales | 21.50% | Fusobacteriales | 7.78% | Bacillales | 7.16% | Bacteroidales | 5.14% |
| Fusobacteriales | 1.87% | Selenomonadales | 3.81% | Mycoplasmatales | 8.05% | Legionellales | 6.16% | Pseudomonadales | 6.98% | Fusobacteriales | 2.48% |
| Erysipelotrichales | 1.70% | Desulfovibrionales | 2.12% | Rhodospirillales | 3.43% | Erysipelotrichales | 4.28% | Rhodospirillales | 3.93% | Pseudomonadales | 1.78% |
| Desulfovibrionales | 1.07% | Deferribacterales | 0.46% | Spirochaetales | 1.99% | Enterobacteriales | 4.17% | Erysipelotrichales | 3.92% | Bacillales | 1.23% |
| Rickettsiales | 1.02% | Verrucomicrobiales | 0.41% | Selenomonadales | 1.26% | Spirochaetales | 2.35% | Burkholderiales | 3.46% | Burkholderiales | 0.96% |
| Bacillales | 0.96% | Bacillales | 0.26% | Victivallales | 0.57% | Desulfovibrionales | 2.17% | Spirochaetales | 1.72% | Erysipelotrichales | 0.77% |
| Deferribacterales | 0.89% | Coriobacteriales | 0.24% | Firmicutes unclassified | 0.52% | Lactobacillales | 1.80% | Verrucomicrobiales | 1.46% | Propionibacteriales | 0.35% |
| Spirochaetales | 0.78% | Spirochaetales | 0.09% | Erysipelotrichales | 0.44% | Mycoplasmatales | 1.37% | Flavobacteriales | 1.27% | Spirochaetales | 0.34% |
| **Class level** |  |  |  |  |  |  |  |  |  |  |  |
| **top10** | **97.90%** |  | **99.62%** |  | **99.45%** |  | **95.22%** |  | **93.61%** |  | **97.86%** |
| **top3** | **89.25%** |  | **92.13%** |  | **86.15%** |  | **72.84%** |  | **69.02%** |  | **90.54%** |
| Clostridia | 44.56% | Bacteroidia | 56.88% | Alphaproteobacteria | 41.44% | Clostridia | 36.18% | Clostridia | 35.70% | Clostridia | 44.36% |
| Bacteroidia | 42.77% | Clostridia | 24.39% | Bacteroidia | 23.21% | Bacteroidia | 25.68% | Bacteroidia | 25.75% | Gammaproteobacteria | 41.04% |
| Negativicutes | 1.92% | Erysipelotrichia | 10.86% | Clostridia | 21.50% | Gammaproteobacteria | 10.98% | Gammaproteobacteria | 7.57% | Bacteroidia | 5.14% |
| Fusobacteriia | 1.87% | Negativicutes | 3.81% | Mollicutes | 8.10% | Fusobacteriia | 7.78% | Bacilli | 7.32% | Fusobacteriia | 2.48% |
| Erysipelotrichia | 1.70% | Deltaproteobacteria | 2.12% | Spirochaetes | 1.99% | Erysipelotrichia | 4.28% | Alphaproteobacteria | 5.16% | Bacilli | 1.30% |
| Bacilli | 1.23% | Deferribacteres | 0.46% | Negativicutes | 1.26% | Spirochaetes | 2.35% | Erysipelotrichia | 3.92% | Betaproteobacteria | 0.98% |
| Alphaproteobacteria | 1.11% | Verrucomicrobiae | 0.41% | Lentisphaeria | 0.57% | Deltaproteobacteria | 2.18% | Betaproteobacteria | 3.67% | Erysipelotrichia | 0.77% |
| Deltaproteobacteria | 1.07% | Bacilli | 0.26% | Firmicutes unclassified | 0.52% | Bacilli | 2.17% | Spirochaetes | 1.74% | Alphaproteobacteria | 0.73% |
| Deferribacteres | 0.89% | Coriobacteriia | 0.24% | Erysipelotrichia | 0.44% | Mollicutes | 1.88% | Verrucomicrobiae | 1.46% | Actinobacteria | 0.72% |
| Spirochaetes | 0.78% | Alphaproteobacteria | 0.19% | Bacilli | 0.42% | Alphaproteobacteria | 1.74% | Actinobacteria | 1.32% | Spirochaetes | 0.34% |
| **Phylum level** |  |  |  |  |  |  |  |  |  |  |  |
| **top10** | **99.81%** |  | **99.99%** |  | **99.93%** |  | **99.12%** |  | **98.48%** |  | **99.62%** |
| **top3** | **95.19%** |  | **98.66%** |  | **88.92%** |  | **84.53%** |  | **91.62%** |  | **95.15%** |
| Firmicutes | 49.68% | Bacteroidetes | 56.90% | Proteobacteria | 41.53% | Firmicutes | 43.69% | Firmicutes | 47.23% | Firmicutes | 46.68% |
| Bacteroidetes | 42.79% | Firmicutes | 39.34% | Firmicutes | 24.14% | Bacteroidetes | 25.73% | Bacteroidetes | 27.22% | Proteobacteria | 43.02% |
| Proteobacteria | 2.72% | Proteobacteria | 2.42% | Bacteroidetes | 23.25% | Proteobacteria | 15.11% | Proteobacteria | 17.17% | Bacteroidetes | 5.45% |
| Fusobacteria | 1.87% | Deferribacteres | 0.46% | Tenericutes | 8.10% | Fusobacteria | 7.78% | Actinobacteria | 1.78% | Fusobacteria | 2.48% |
| Deferribacteres | 0.89% | Verrucomicrobia | 0.41% | Spirochaetae | 1.99% | Spirochaetae | 2.35% | Spirochaetae | 1.74% | Actinobacteria | 0.87% |
| Spirochaetae | 0.78% | Actinobacteria | 0.28% | Lentisphaerae | 0.57% | Tenericutes | 1.88% | Verrucomicrobia | 1.48% | Verrucomicrobia | 0.36% |
| Actinobacteria | 0.39% | Spirochaetae | 0.09% | Cyanobacteria | 0.19% | Verrucomicrobia | 1.24% | Tenericutes | 0.73% | Spirochaetae | 0.34% |
| Cyanobacteria | 0.26% | Bacteria unclassified | 0.07% | Actinobacteria | 0.08% | Actinobacteria | 0.56% | Deinococcus-Thermus | 0.39% | Bacteria unclassified | 0.18% |
| Verrucomicrobia | 0.24% | Acidobacteria | 0.01% | Deferribacteres | 0.04% | Deferribacteres | 0.47% | Lentisphaerae | 0.38% | Deinococcus-Thermus | 0.12% |
| Tenericutes | 0.19% | Tenericutes | 0.01% | Verrucomicrobia | 0.04% | Lentisphaerae | 0.31% | Bacteria unclassified | 0.36% | Tenericutes | 0.12% |

**Table S3** List of categories of the habitat generalists, specialists and too-rare types at the phylum level.

| Phylum | Natural Habitat | |  | Farmland |  | Classes |
| --- | --- | --- | --- | --- | --- | --- |
|  | Number of reads | Proportion |  | Number of reads | Proportion |  |
| Firmicutes | 20019 | 33.06% |  | 25324 | 46.02% | Generalist |
| Bacteroidetes | 21460 | 35.44% |  | 10734 | 19.51% | Generalist |
| Proteobacteria | 14580 | 24.08% |  | 13876 | 25.22% | Generalist |
| Spirochaetae | 775 | 1.28% |  | 797 | 1.45% | Generalist |
| Lentisphaerae | 192 | 0.32% |  | 136 | 0.25% | Generalist |
| Deferribacteres | 191 | 0.32% |  | 88 | 0.16% | Generalist |
| Cyanobacteria | 95 | 0.16% |  | 136 | 0.25% | Generalist |
| Planctomycetes | 17 | 0.03% |  | 46 | 0.08% | Generalist |
| Fusobacteria | 235 | 0.39% |  | 1728 | 3.14% | Specialist_F |
| Actinobacteria | 116 | 0.19% |  | 617 | 1.12% | Specialist_F |
| Verrucomicrobia | 102 | 0.17% |  | 571 | 1.04% | Specialist_F |
| Bacteria_unclassified | 20 | 0.03% |  | 151 | 0.27% | Specialist_F |
| Deinococcus.Thermus | 4 | 0.01% |  | 105 | 0.19% | Specialist_F |
| Acidobacteria | 8 | 0.01% |  | 94 | 0.17% | Specialist_F |
| Elusimicrobia | 0 | <0.01% |  | 49 | 0.09% | Specialist_F |
| Synergistetes | 0 | <0.01% |  | 33 | 0.06% | Specialist_F |
| Chloroflexi | 0 | <0.01% |  | 22 | 0.04% | Specialist_F |
| Gemmatimonadetes | 1 | <0.01% |  | 14 | 0.03% | Specialist_F |
| Tenericutes | 2736 | 4.52% |  | 473 | 0.86% | Specialist_N |
| Nitrospirae | 1 | <0.01% |  | 11 | 0.02% | Too_rare |
| Candidate_division_TM7 | 0 | <0.01% |  | 7 | 0.01% | Too_rare |
| Candidate_division_WS3 | 2 | <0.01% |  | 5 | 0.01% | Too_rare |
| Armatimonadetes | 2 | <0.01% |  | 1 | <0.01% | Too_rare |
| Chlorobi | 0 | <0.01% |  | 2 | <0.01% | Too_rare |
| Fibrobacteres | 0 | <0.01% |  | 2 | <0.01% | Too_rare |
| RsaHF231 | 2 | <0.01% |  | 0 | <0.01% | Too_rare |
| BHI80.139 | 0 | <0.01% |  | 1 | <0.01% | Too_rare |
| Candidate_division_OP3 | 0 | <0.01% |  | 1 | <0.01% | Too_rare |
| SM2F11 | 0 | <0.01% |  | 1 | <0.01% | Too_rare |

**Table S4** List of categories of the habitat generalists, specialists and too-rare types at the species level.

| Species | Natural Habitat | |  | Farmland | | Classes |
| --- | --- | --- | --- | --- | --- | --- |
|  | Number of reads | Proportion |  | Number of reads | Proportion |  |
| Lachnospiraceae_unclassified | 7958 | 13.14% |  | 4679 | 8.50% | Generalist |
| Bacteroides_unclassified | 6885 | 11.37% |  | 4144 | 7.53% | Generalist |
| Ruminococcaceae_unclassified | 3375 | 5.57% |  | 3642 | 6.62% | Generalist |
| Anaerotruncus_unclassified | 531 | 0.88% |  | 909 | 1.65% | Generalist |
| Alistipes_unclassified | 1680 | 2.77% |  | 698 | 1.27% | Generalist |
| Odoribacter_uncultured_bacterium | 409 | 0.68% |  | 514 | 0.93% | Generalist |
| Thalassospira_unclassified | 429 | 0.71% |  | 438 | 0.80% | Generalist |
| Clostridiales_unclassified | 358 | 0.59% |  | 392 | 0.71% | Generalist |
| Odoribacter_unclassified | 600 | 0.99% |  | 387 | 0.70% | Generalist |
| Eubacterium_dolichum | 174 | 0.29% |  | 306 | 0.56% | Generalist |
| Desulfovibrio_unclassified | 367 | 0.61% |  | 253 | 0.46% | Generalist |
| Porphyromonadaceae_unclassified | 264 | 0.44% |  | 237 | 0.43% | Generalist |
| Eubacterium_fissicatena | 297 | 0.49% |  | 210 | 0.38% | Generalist |
| Anaerorhabdus_furcosa | 399 | 0.66% |  | 196 | 0.36% | Generalist |
| Bacteroidales_unclassified | 166 | 0.27% |  | 184 | 0.33% | Generalist |
| Anaerotruncus_uncultured_bacterium | 225 | 0.37% |  | 118 | 0.21% | Generalist |
| Desulfovibrionaceae_unclassified | 42 | 0.07% |  | 94 | 0.17% | Generalist |
| Ruminococcaceae_uncultured_bacterium | 134 | 0.22% |  | 89 | 0.16% | Generalist |
| vadinBB60_unclassified | 51 | 0.08% |  | 77 | 0.14% | Generalist |
| Rikenella_microfusus | 138 | 0.23% |  | 74 | 0.13% | Generalist |
| Peptococcaceae_unclassified | 43 | 0.07% |  | 62 | 0.11% | Generalist |
| Massilia_alkalitolerans | 17 | 0.03% |  | 45 | 0.08% | Generalist |
| Coriobacteriaceae_unclassified | 30 | 0.05% |  | 44 | 0.08% | Generalist |
| Paenibacillus_sp._7.5 | 26 | 0.04% |  | 43 | 0.08% | Generalist |
| Hydrogenoanaerobacterium_unclassified | 79 | 0.13% |  | 41 | 0.07% | Generalist |
| Butyricimonas_unclassified | 73 | 0.12% |  | 38 | 0.07% | Generalist |
| Christensenellaceae_uncultured_bacterium | 17 | 0.03% |  | 35 | 0.06% | Generalist |
| Tannerella_sp._6_1_58FAA_CT1 | 31 | 0.05% |  | 32 | 0.06% | Generalist |
| Bacteroidetes_unclassified | 11 | 0.02% |  | 26 | 0.05% | Generalist |
| Barnesiella_uncultured_porphyromonadaceae_bacterium | 35 | 0.06% |  | 24 | 0.04% | Generalist |
| Candidatus_Captivus_uncultured_alpha_proteobacterium | 84 | 0.14% |  | 23 | 0.04% | Generalist |
| Subgroup_6_unclassified | 4 | 0.01% |  | 19 | 0.03% | Generalist |
| Christensenella_minuta | 9 | 0.01% |  | 18 | 0.03% | Generalist |
| Chloroplast_unclassified | 17 | 0.03% |  | 16 | 0.03% | Generalist |
| Ochrobactrum_uncultured_ochrobactrum_sp. | 3 | <0.01% |  | 16 | 0.03% | Generalist |
| Brevundimonas_unclassified | 2 | <0.01% |  | 14 | 0.03% | Generalist |
| Alistipes_uncultured_rikenellaceae_bacterium | 23 | 0.04% |  | 13 | 0.02% | Generalist |
| Alistipes_uncultured_bacteroidaceae_bacterium | 46 | 0.08% |  | 12 | 0.02% | Generalist |
| Oxalobacter_formigenes | 7 | 0.01% |  | 12 | 0.02% | Generalist |
| Xanthomonadales_unclassified | 2 | <0.01% |  | 12 | 0.02% | Generalist |
| Rikenella_uncultured_rikenella_sp. | 21 | 0.03% |  | 10 | 0.02% | Generalist |
| Methylobacterium_adhaesivum | 7 | 0.01% |  | 9 | 0.02% | Generalist |
| Gordonibacter_uncultured_bacterium | 20 | 0.03% |  | 4 | 0.01% | Generalist |
| MLE1.12_uncultured_bacterium | 16 | 0.03% |  | 2 | <0.01% | Generalist |
| Morganella_unclassified | 0 | <0.01% |  | 6026 | 10.95% | Specialist_F |
| Peptostreptococcaceae_unclassified | 81 | 0.13% |  | 2800 | 5.09% | Specialist_F |
| Clostridium_perfringens | 0 | <0.01% |  | 2633 | 4.79% | Specialist_F |
| Lachnospiraceae_uncultured_bacterium | 473 | 0.78% |  | 1818 | 3.30% | Specialist_F |
| Cetobacterium_uncultured_bacterium | 2 | <0.01% |  | 1723 | 3.13% | Specialist_F |
| Brevibacillus_agri | 101 | 0.17% |  | 1477 | 2.68% | Specialist_F |
| Morganella_uncultured_morganella_sp. | 0 | <0.01% |  | 1262 | 2.29% | Specialist_F |
| Alistipes_uncultured_bacterium | 92 | 0.15% |  | 1173 | 2.13% | Specialist_F |
| Clostridium_uncultured_bacterium | 58 | 0.10% |  | 1133 | 2.06% | Specialist_F |
| Legionella_sp. | 0 | <0.01% |  | 987 | 1.79% | Specialist_F |
| Erysipelotrichaceae_unclassified | 257 | 0.42% |  | 933 | 1.70% | Specialist_F |
| Pseudomonas_unclassified | 6 | 0.01% |  | 861 | 1.56% | Specialist_F |
| Clostridium_baratii | 24 | 0.04% |  | 759 | 1.38% | Specialist_F |
| Treponema_unclassified | 50 | 0.08% |  | 705 | 1.28% | Specialist_F |
| Acinetobacter_unclassified | 5 | 0.01% |  | 629 | 1.14% | Specialist_F |
| Akkermansia_unclassified | 97 | 0.16% |  | 546 | 0.99% | Specialist_F |
| Roseomonas_unclassified | 4 | 0.01% |  | 540 | 0.98% | Specialist_F |
| Delftia_uncultured_bacterium | 2 | <0.01% |  | 494 | 0.90% | Specialist_F |
| Citrobacter_unclassified | 1 | <0.01% |  | 492 | 0.89% | Specialist_F |
| Oscillibacter_unclassified | 52 | 0.09% |  | 420 | 0.76% | Specialist_F |
| Acinetobacter_schindleri | 14 | 0.02% |  | 322 | 0.59% | Specialist_F |
| Barnesiella_uncultured_bacterium | 0 | <0.01% |  | 304 | 0.55% | Specialist_F |
| butyrate.producing_bacterium_A2.207 | 1 | <0.01% |  | 302 | 0.55% | Specialist_F |
| Chryseobacterium_unclassified | 1 | <0.01% |  | 286 | 0.52% | Specialist_F |
| Subdoligranulum_sp._4_3_54A2FAA | 88 | 0.15% |  | 282 | 0.51% | Specialist_F |
| Enterococcus_gilvus | 15 | 0.02% |  | 270 | 0.49% | Specialist_F |
| Mycoplasma_unclassified | 2 | <0.01% |  | 262 | 0.48% | Specialist_F |
| Flavonifractor_uncultured_bacterium | 21 | 0.03% |  | 251 | 0.46% | Specialist_F |
| Bilophila_uncultured_bacterium | 45 | 0.07% |  | 156 | 0.28% | Specialist_F |
| X.Clostridium._bifermentans | 10 | 0.02% |  | 155 | 0.28% | Specialist_F |
| Pelomonas_uncultured_bacterium | 3 | <0.01% |  | 155 | 0.28% | Specialist_F |
| Propionibacterium_unclassified | 10 | 0.02% |  | 154 | 0.28% | Specialist_F |
| Bacteria_unclassified | 20 | 0.03% |  | 151 | 0.27% | Specialist_F |
| Victivallaceae_unclassified | 0 | <0.01% |  | 122 | 0.22% | Specialist_F |
| X4C0d.2_uncultured_bacterium | 21 | 0.03% |  | 112 | 0.20% | Specialist_F |
| Gordonibacter_unclassified | 16 | 0.03% |  | 106 | 0.19% | Specialist_F |
| Enterobacteriaceae_unclassified | 1 | <0.01% |  | 96 | 0.17% | Specialist_F |
| RF9_uncultured_bacterium | 12 | 0.02% |  | 87 | 0.16% | Specialist_F |
| Thermus_unclassified | 3 | <0.01% |  | 87 | 0.16% | Specialist_F |
| Burkholderia_uncultured_beta_proteobacterium | 0 | <0.01% |  | 76 | 0.14% | Specialist_F |
| RF9_unclassified | 2 | <0.01% |  | 72 | 0.13% | Specialist_F |
| Alphaproteobacteria_unclassified | 0 | <0.01% |  | 72 | 0.13% | Specialist_F |
| Macellibacteroides_uncultured_bacterium | 0 | <0.01% |  | 66 | 0.12% | Specialist_F |
| Acidovorax_citrulli | 17 | 0.03% |  | 60 | 0.11% | Specialist_F |
| Paenibacillus_sp._J16.10 | 15 | 0.02% |  | 59 | 0.11% | Specialist_F |
| Barnesiella_unclassified | 0 | <0.01% |  | 58 | 0.11% | Specialist_F |
| vadinBB60_uncultured_bacterium | 1 | <0.01% |  | 55 | 0.10% | Specialist_F |
| Moraxella_osloensis | 0 | <0.01% |  | 55 | 0.10% | Specialist_F |
| Nocardioides_uncultured_bacterium | 3 | <0.01% |  | 53 | 0.10% | Specialist_F |
| Brevinema_andersonii | 9 | 0.01% |  | 51 | 0.09% | Specialist_F |
| Corynebacterium_unclassified | 3 | <0.01% |  | 50 | 0.09% | Specialist_F |
| Sphingomonas_unclassified | 2 | <0.01% |  | 50 | 0.09% | Specialist_F |
| Elusimicrobium_uncultured_rumen_bacterium_4c0d.3 | 0 | <0.01% |  | 48 | 0.09% | Specialist_F |
| Acholeplasma_unclassified | 7 | 0.01% |  | 46 | 0.08% | Specialist_F |
| Corynebacterium_mycetoides | 0 | <0.01% |  | 42 | 0.08% | Specialist_F |
| Paenibacillus_unclassified | 7 | 0.01% |  | 38 | 0.07% | Specialist_F |
| Bacillus_unclassified | 2 | <0.01% |  | 34 | 0.06% | Specialist_F |
| Deferribacteraceae_unclassified | 0 | <0.01% |  | 34 | 0.06% | Specialist_F |
| Ruminococcaceae_uncultured_pseudoflavonifractor_sp. | 6 | 0.01% |  | 33 | 0.06% | Specialist_F |
| Synergistaceae_unclassified | 0 | <0.01% |  | 32 | 0.06% | Specialist_F |
| Enterobacter_sp._638 | 0 | <0.01% |  | 31 | 0.06% | Specialist_F |
| Lysinibacillus_unclassified | 0 | <0.01% |  | 29 | 0.05% | Specialist_F |
| Stenotrophomonas_uncultured_stenotrophomonas_sp. | 0 | <0.01% |  | 27 | 0.05% | Specialist_F |
| Cupriavidus_unclassified | 0 | <0.01% |  | 27 | 0.05% | Specialist_F |
| Staphylococcus_epidermidis_RP62A_phage_SP.beta | 2 | <0.01% |  | 24 | 0.04% | Specialist_F |
| Corynebacterium_tuberculostearicum | 2 | <0.01% |  | 22 | 0.04% | Specialist_F |
| bacterium_.Lincoln_Park_3. | 2 | <0.01% |  | 22 | 0.04% | Specialist_F |
| Methylobacterium_radiotolerans | 0 | <0.01% |  | 20 | 0.04% | Specialist_F |
| Sphingomonadaceae_unclassified | 0 | <0.01% |  | 20 | 0.04% | Specialist_F |
| Dermacoccus_uncultured_bacterium | 0 | <0.01% |  | 20 | 0.04% | Specialist_F |
| Nitrosomonadaceae_uncultured_burkholderiaceae_bacterium | 0 | <0.01% |  | 19 | 0.03% | Specialist_F |
| Methylobacterium_unclassified | 1 | <0.01% |  | 18 | 0.03% | Specialist_F |
| S24.7_uncultured_bacterium | 0 | <0.01% |  | 17 | 0.03% | Specialist_F |
| endosymbiont_.TC1._of_Trimyema_compressum | 0 | <0.01% |  | 17 | 0.03% | Specialist_F |
| Rhodococcus_unclassified | 0 | <0.01% |  | 16 | 0.03% | Specialist_F |
| Flavobacterium_unclassified | 0 | <0.01% |  | 16 | 0.03% | Specialist_F |
| Shinella_uncultured_bacterium | 1 | <0.01% |  | 15 | 0.03% | Specialist_F |
| Paenibacillus_taiwanensis | 0 | <0.01% |  | 15 | 0.03% | Specialist_F |
| Meiothermus_silvanus_DSM_9946 | 0 | <0.01% |  | 15 | 0.03% | Specialist_F |
| Bacillus_oceanisediminis | 0 | <0.01% |  | 15 | 0.03% | Specialist_F |
| Caulobacteraceae_unclassified | 0 | <0.01% |  | 13 | 0.02% | Specialist_F |
| Rickettsiella_sp._GSU | 0 | <0.01% |  | 13 | 0.02% | Specialist_F |
| Candidatus_Hepatincola_uncultured_alpha_proteobacterium | 12731 | 21.02% |  | 43 | 0.08% | Specialist_N |
| Parabacteroides_unclassified | 6399 | 10.57% |  | 1585 | 2.88% | Specialist_N |
| Ureaplasma_unclassified | 2713 | 4.48% |  | 1 | 0.00% | Specialist_N |
| Clostridium_colicanis | 1406 | 2.32% |  | 121 | 0.22% | Specialist_N |
| Coprobacillus_uncultured_bacterium | 1092 | 1.80% |  | 196 | 0.36% | Specialist_N |
| Bacteroides_uncultured_bacterium | 1091 | 1.80% |  | 100 | 0.18% | Specialist_N |
| Rikenella_unclassified | 977 | 1.61% |  | 26 | 0.05% | Specialist_N |
| Rikenella_uncultured_bacterium | 918 | 1.52% |  | 345 | 0.63% | Specialist_N |
| Rikenellaceae_unclassified | 907 | 1.50% |  | 188 | 0.34% | Specialist_N |
| Veillonellaceae_unclassified | 821 | 1.36% |  | 207 | 0.38% | Specialist_N |
| Brachyspira_unclassified | 716 | 1.18% |  | 33 | 0.06% | Specialist_N |
| Rhodospirillaceae_uncultured_rumen_bacterium | 713 | 1.18% |  | 1 | 0.00% | Specialist_N |
| Parabacteroides_uncultured_bacterium | 522 | 0.86% |  | 113 | 0.21% | Specialist_N |
| Clostridium_sp._YIT_12070 | 390 | 0.64% |  | 104 | 0.19% | Specialist_N |
| Succinispira_uncultured_bacterium | 355 | 0.59% |  | 9 | 0.02% | Specialist_N |
| Clostridium_unclassified | 279 | 0.46% |  | 35 | 0.06% | Specialist_N |
| Fusobacterium_varium | 233 | 0.38% |  | 0 | 0.00% | Specialist_N |
| Firmicutes_unclassified | 209 | 0.35% |  | 57 | 0.10% | Specialist_N |
| Victivallis_unclassified | 191 | 0.32% |  | 11 | 0.02% | Specialist_N |
| Mucispirillum_unclassified | 189 | 0.31% |  | 32 | 0.06% | Specialist_N |
| bacterium_enrichment_culture_clone_Ecwsrb033 | 130 | 0.21% |  | 5 | 0.01% | Specialist_N |
| Alistipes_indistinctus_YIT_12060 | 86 | 0.14% |  | 9 | 0.02% | Specialist_N |
| Enterococcus_unclassified | 81 | 0.13% |  | 15 | 0.03% | Specialist_N |
| Lactobacillus_gasseri | 62 | 0.10% |  | 6 | 0.01% | Specialist_N |
| S24.7_uncultured_bacteroidales_bacterium | 45 | 0.07% |  | 3 | 0.01% | Specialist_N |
| Acidaminococcaceae_uncultured_veillonellaceae_bacterium | 44 | 0.07% |  | 0 | <0.01% | Specialist_N |
| X4C0d.2_unclassified | 34 | 0.06% |  | 3 | 0.01% | Specialist_N |
| Clostridium_propionicum | 30 | 0.05% |  | 2 | <0.01% | Specialist_N |
| Clostridium_saccharogumia | 29 | 0.05% |  | 0 | <0.01% | Specialist_N |
| Clostridium_hathewayi | 27 | 0.04% |  | 1 | <0.01% | Specialist_N |
| Clostridiales_uncultured_bacterium | 25 | 0.04% |  | 0 | <0.01% | Specialist_N |
| Bacteroides_uncultured_bacteroidaceae_bacterium | 24 | 0.04% |  | 2 | <0.01% | Specialist_N |
| Clostridium_methylpentosum_DSM_5476 | 19 | 0.03% |  | 0 | <0.01% | Specialist_N |
| Clostridiaceae_bacterium_FH052 | 14 | 0.02% |  | 1 | <0.01% | Specialist_N |
| Sphingomonadales_unclassified | 0 | <0.01% |  | 12 | 0.02% | Too_rare |
| vadinHA64_uncultured_rumen_bacterium | 0 | <0.01% |  | 12 | 0.02% | Too_rare |
| Rhodospirillaceae_unclassified | 0 | <0.01% |  | 11 | 0.02% | Too_rare |
| Sedimentibacter_hongkongensis | 0 | <0.01% |  | 11 | 0.02% | Too_rare |
| Thalassospira_uncultured_bacterium | 3 | <0.01% |  | 10 | 0.02% | Too_rare |
| Blastocatella_unclassified | 1 | <0.01% |  | 10 | 0.02% | Too_rare |
| RB41_unclassified | 0 | <0.01% |  | 10 | 0.02% | Too_rare |
| Gaiellales_unclassified | 0 | <0.01% |  | 10 | 0.02% | Too_rare |
| Haliangium_unclassified | 0 | <0.01% |  | 10 | 0.02% | Too_rare |
| Sedimentibacter_unclassified | 3 | <0.01% |  | 9 | 0.02% | Too_rare |
| Flavonifractor_unclassified | 1 | <0.01% |  | 9 | 0.02% | Too_rare |
| Eubacterium_limosum | 1 | <0.01% |  | 9 | 0.02% | Too_rare |
| Xanthobacteraceae_unclassified | 1 | <0.01% |  | 9 | 0.02% | Too_rare |
| vadinBB60_uncultured_catabacteraceae_bacterium | 0 | <0.01% |  | 9 | 0.02% | Too_rare |
| Brevundimonas_vesicularis | 3 | <0.01% |  | 8 | 0.01% | Too_rare |
| Clostridiaceae_bacterium_FN062 | 2 | <0.01% |  | 8 | 0.01% | Too_rare |
| Enterococcus_gallinarum | 1 | <0.01% |  | 8 | 0.01% | Too_rare |
| Lactococcus_unclassified | 0 | <0.01% |  | 8 | 0.01% | Too_rare |
| Kluyvera_ascorbata | 0 | <0.01% |  | 8 | 0.01% | Too_rare |
| Epulopiscium_uncultured_bacterium | 0 | <0.01% |  | 8 | 0.01% | Too_rare |
| Methylobacterium_hispanicum | 0 | <0.01% |  | 8 | 0.01% | Too_rare |
| Sporolactobacillus_uncultured_organism | 0 | <0.01% |  | 8 | 0.01% | Too_rare |
| Peptococcaceae_uncultured_bacterium | 0 | <0.01% |  | 8 | 0.01% | Too_rare |
| KF.JG30.B3_uncultured_bacterium | 0 | <0.01% |  | 8 | 0.01% | Too_rare |
| Bdellovibrio_bacteriovorus | 0 | <0.01% |  | 8 | 0.01% | Too_rare |
| WD2101_soil_group_uncultured_bacterium | 0 | <0.01% |  | 8 | 0.01% | Too_rare |
| Lactobacillus_unclassified | 6 | 0.01% |  | 7 | 0.01% | Too_rare |
| unidentified_rumen_bacterium_JW32 | 4 | 0.01% |  | 7 | 0.01% | Too_rare |
| Massilia_unclassified | 2 | <0.01% |  | 7 | 0.01% | Too_rare |
| Kocuria_unclassified | 0 | <0.01% |  | 7 | 0.01% | Too_rare |
| Stenotrophomonas_unclassified | 0 | <0.01% |  | 7 | 0.01% | Too_rare |
| Holosporaceae_uncultured_rickettsiales_bacterium | 0 | <0.01% |  | 7 | 0.01% | Too_rare |
| Roseiflexus_unclassified | 0 | <0.01% |  | 7 | 0.01% | Too_rare |
| Ruminococcaceae_uncultured_rumen_bacterium_4c28d.12 | 7 | 0.01% |  | 6 | 0.01% | Too_rare |
| Blautia_unclassified | 3 | <0.01% |  | 6 | 0.01% | Too_rare |
| Acidimicrobiales_unclassified | 3 | <0.01% |  | 6 | 0.01% | Too_rare |
| Rhizobium_unclassified | 1 | <0.01% |  | 6 | 0.01% | Too_rare |
| Novosphingobium_unclassified | 1 | <0.01% |  | 6 | 0.01% | Too_rare |
| Massilia_timonae | 0 | <0.01% |  | 6 | 0.01% | Too_rare |
| Sphingomonas_koreensis | 0 | <0.01% |  | 6 | 0.01% | Too_rare |
| Nevskia_unclassified | 0 | <0.01% |  | 6 | 0.01% | Too_rare |
| Escherichia.Shigella_unclassified | 0 | <0.01% |  | 6 | 0.01% | Too_rare |
| Micrococcus_luteus | 0 | <0.01% |  | 6 | 0.01% | Too_rare |
| Subgroup_6_uncultured_acidobacteria_bacterium | 0 | <0.01% |  | 6 | 0.01% | Too_rare |
| Sphingomonas_melonis | 0 | <0.01% |  | 6 | 0.01% | Too_rare |
| Undibacterium_uncultured_bacterium | 0 | <0.01% |  | 6 | 0.01% | Too_rare |
| Facklamia_uncultured_bacterium | 0 | <0.01% |  | 6 | 0.01% | Too_rare |
| Bilophila_unclassified | 0 | <0.01% |  | 6 | 0.01% | Too_rare |
| vadinHA64_unclassified | 0 | <0.01% |  | 6 | 0.01% | Too_rare |
| SM1A02_uncultured_bacterium | 0 | <0.01% |  | 6 | 0.01% | Too_rare |
| mitochondria_uncultured_alpha_proteobacterium | 9 | 0.01% |  | 5 | 0.01% | Too_rare |
| Bradyrhizobium_unclassified | 4 | 0.01% |  | 5 | 0.01% | Too_rare |
| Exiguobacterium_mexicanum | 4 | 0.01% |  | 5 | 0.01% | Too_rare |
| Mesorhizobium_uncultured_mesorhizobium_sp. | 1 | <0.01% |  | 5 | 0.01% | Too_rare |
| Lactobacillus_casei | 1 | <0.01% |  | 5 | 0.01% | Too_rare |
| unidentified_rumen_bacterium_12.110 | 1 | <0.01% |  | 5 | 0.01% | Too_rare |
| Candidate_division_TM7_unclassified | 0 | <0.01% |  | 5 | 0.01% | Too_rare |
| Hados.Sed.Eubac.3_uncultured_bacterium | 0 | <0.01% |  | 5 | 0.01% | Too_rare |
| Staphylococcus_saprophyticus_subsp._saprophyticus_ATCC_15305 | 0 | <0.01% |  | 5 | 0.01% | Too_rare |
| Bordetella_sp._BF07B02 | 0 | <0.01% |  | 5 | 0.01% | Too_rare |
| Bacillus_firmus | 0 | <0.01% |  | 5 | 0.01% | Too_rare |
| Gemmatimonadaceae_uncultured_bacterium | 0 | <0.01% |  | 5 | 0.01% | Too_rare |
| Subgroup_6_uncultured_bacterium | 0 | <0.01% |  | 5 | 0.01% | Too_rare |
| RB41_uncultured_acidobacteria_bacterium | 0 | <0.01% |  | 5 | 0.01% | Too_rare |
| Reyranella_uncultured_alpha_proteobacterium | 0 | <0.01% |  | 5 | 0.01% | Too_rare |
| Devosia_uncultured_bacterium | 0 | <0.01% |  | 5 | 0.01% | Too_rare |
| Mycoplasmataceae_unclassified | 0 | <0.01% |  | 5 | 0.01% | Too_rare |
| Wolbachia_uncultured_alpha_proteobacterium | 0 | <0.01% |  | 5 | 0.01% | Too_rare |
| Planctomycetaceae_uncultured_bacterium | 5 | 0.01% |  | 4 | 0.01% | Too_rare |
| Microbacterium_paraoxydans | 3 | <0.01% |  | 4 | 0.01% | Too_rare |
| Proteobacteria_unclassified | 2 | <0.01% |  | 4 | 0.01% | Too_rare |
| Propionibacterium_granulosum | 2 | <0.01% |  | 4 | 0.01% | Too_rare |
| Pseudacidovorax_unclassified | 2 | <0.01% |  | 4 | 0.01% | Too_rare |
| Brachybacterium_unclassified | 1 | <0.01% |  | 4 | 0.01% | Too_rare |
| Ralstonia_unclassified | 1 | <0.01% |  | 4 | 0.01% | Too_rare |
| Christensenella_unclassified | 1 | <0.01% |  | 4 | 0.01% | Too_rare |
| Phenylobacterium_unclassified | 1 | <0.01% |  | 4 | 0.01% | Too_rare |
| Subgroup_17_uncultured_bacterium | 1 | <0.01% |  | 4 | 0.01% | Too_rare |
| Lactobacillales_bacterium_HY.36.1 | 0 | <0.01% |  | 4 | 0.01% | Too_rare |
| Desulfovibrio_uncultured_bacterium | 0 | <0.01% |  | 4 | 0.01% | Too_rare |
| Spirochaetaceae_unclassified | 0 | <0.01% |  | 4 | 0.01% | Too_rare |
| TRA3.20_unclassified | 0 | <0.01% |  | 4 | 0.01% | Too_rare |
| Bacillus_arsenicus | 0 | <0.01% |  | 4 | 0.01% | Too_rare |
| Chitinophagaceae_unclassified | 0 | <0.01% |  | 4 | 0.01% | Too_rare |
| X0319.6A21_uncultured_bacterium | 0 | <0.01% |  | 4 | 0.01% | Too_rare |
| GR.WP33.30_unclassified | 0 | <0.01% |  | 4 | 0.01% | Too_rare |
| Turicibacter_unclassified | 0 | <0.01% |  | 4 | 0.01% | Too_rare |
| X0319.6G20_unclassified | 0 | <0.01% |  | 4 | 0.01% | Too_rare |
| Legionella_unclassified | 0 | <0.01% |  | 4 | 0.01% | Too_rare |
| Legionella_uncultured_bacterium | 0 | <0.01% |  | 4 | 0.01% | Too_rare |
| Plesiomonas_uncultured_bacterium | 0 | <0.01% |  | 4 | 0.01% | Too_rare |
| SHA.4_uncultured_bacterium | 0 | <0.01% |  | 4 | 0.01% | Too_rare |
| Spirosoma_unclassified | 0 | <0.01% |  | 4 | 0.01% | Too_rare |
| Acidimicrobiales_uncultured_forest_soil_bacterium | 9 | 0.01% |  | 3 | 0.01% | Too_rare |
| Clostridium_sartagoforme | 5 | 0.01% |  | 3 | 0.01% | Too_rare |
| Gemmata_uncultured_bacterium | 5 | 0.01% |  | 3 | 0.01% | Too_rare |
| Victivallis_uncultured_bacterium | 1 | <0.01% |  | 3 | 0.01% | Too_rare |
| Bosea_thiooxidans | 1 | <0.01% |  | 3 | 0.01% | Too_rare |
| Pseudomonas_otitidis | 1 | <0.01% |  | 3 | 0.01% | Too_rare |
| Rhodobacteraceae_unclassified | 1 | <0.01% |  | 3 | 0.01% | Too_rare |
| TRA3.20_uncultured_beta_proteobacterium | 1 | <0.01% |  | 3 | 0.01% | Too_rare |
| Microbacterium_flavescens | 1 | <0.01% |  | 3 | 0.01% | Too_rare |
| Dysgonomonas_uncultured_bacterium | 0 | <0.01% |  | 3 | 0.01% | Too_rare |
| Emticicia_sp._IMCC1731 | 0 | <0.01% |  | 3 | 0.01% | Too_rare |
| Sphingobacterium_multivorum | 0 | <0.01% |  | 3 | 0.01% | Too_rare |
| Dysgonomonas_uncultured_dysgonomonas_sp. | 0 | <0.01% |  | 3 | 0.01% | Too_rare |
| Microbacterium_unclassified | 0 | <0.01% |  | 3 | 0.01% | Too_rare |
| SC.I.84_uncultured_bacterium | 0 | <0.01% |  | 3 | 0.01% | Too_rare |
| Sphingobacterium_composti_Yoo_et_al._2007 | 0 | <0.01% |  | 3 | 0.01% | Too_rare |
| Acidovorax_wohlfahrtii | 0 | <0.01% |  | 3 | 0.01% | Too_rare |
| Anaerolineaceae_uncultured_soil_bacterium | 0 | <0.01% |  | 3 | 0.01% | Too_rare |
| Planctomycetaceae_unclassified | 0 | <0.01% |  | 3 | 0.01% | Too_rare |
| Planococcaceae_unclassified | 0 | <0.01% |  | 3 | 0.01% | Too_rare |
| Nitrosomonadaceae_unclassified | 0 | <0.01% |  | 3 | 0.01% | Too_rare |
| Chthoniobacter_unclassified | 0 | <0.01% |  | 3 | 0.01% | Too_rare |
| Kocuria_palustris | 0 | <0.01% |  | 3 | 0.01% | Too_rare |
| Subgroup_5_unclassified | 0 | <0.01% |  | 3 | 0.01% | Too_rare |
| Acidobacteriaceae_Subgroup_1_unclassified | 0 | <0.01% |  | 3 | 0.01% | Too_rare |
| CL500.29_marine_group_uncultured_bacterium | 0 | <0.01% |  | 3 | 0.01% | Too_rare |
| Nitrospira_uncultured_nitrospiraceae_bacterium | 0 | <0.01% |  | 3 | 0.01% | Too_rare |
| X480.2_uncultured_actinobacterium | 0 | <0.01% |  | 3 | 0.01% | Too_rare |
| Blastocatella_uncultured_acidobacteria_bacterium | 0 | <0.01% |  | 3 | 0.01% | Too_rare |
| Candidate_division_WS3_uncultured_bacterium | 0 | <0.01% |  | 3 | 0.01% | Too_rare |
| Chloroflexi_unclassified | 0 | <0.01% |  | 3 | 0.01% | Too_rare |
| Corynebacteriaceae_uncultured_bacterium | 0 | <0.01% |  | 3 | 0.01% | Too_rare |
| DB1.14_uncultured_eubacterium_wd2107 | 0 | <0.01% |  | 3 | 0.01% | Too_rare |
| JG34.KF.361_uncultured_bacterium | 0 | <0.01% |  | 3 | 0.01% | Too_rare |
| Opitutus_uncultured_bacterium | 0 | <0.01% |  | 3 | 0.01% | Too_rare |
| PeM15_unclassified | 0 | <0.01% |  | 3 | 0.01% | Too_rare |
| Propionimicrobium_uncultured_bacterium | 0 | <0.01% |  | 3 | 0.01% | Too_rare |
| Rhodocyclaceae_unclassified | 0 | <0.01% |  | 3 | 0.01% | Too_rare |
| SAR202_clade_uncultured_bacterium | 0 | <0.01% |  | 3 | 0.01% | Too_rare |
| SJA.149_uncultured_bacterium | 0 | <0.01% |  | 3 | 0.01% | Too_rare |
| Subgroup_7_uncultured_acidobacteria_bacterium | 0 | <0.01% |  | 3 | 0.01% | Too_rare |
| Reyranella_uncultured_bacterium | 5 | 0.01% |  | 2 | 0.00% | Too_rare |
| Sphingobium_unclassified | 2 | <0.01% |  | 2 | 0.00% | Too_rare |
| Candidate_division_WS3_unclassified | 2 | <0.01% |  | 2 | 0.00% | Too_rare |
| Mycobacterium_unclassified | 2 | <0.01% |  | 2 | 0.00% | Too_rare |
| Gemmatimonadaceae_unclassified | 1 | <0.01% |  | 2 | 0.00% | Too_rare |
| Microbacterium_lacus | 1 | <0.01% |  | 2 | 0.00% | Too_rare |
| Neisseria_uncultured_bacterium | 1 | <0.01% |  | 2 | 0.00% | Too_rare |
| Sphingobacterium_sp._DSM_22361 | 1 | <0.01% |  | 2 | 0.00% | Too_rare |
| Betaproteobacteria_unclassified | 1 | <0.01% |  | 2 | 0.00% | Too_rare |
| Sphingobacterium_uncultured_flavobacterium_sp. | 1 | <0.01% |  | 2 | <0.01% | Too_rare |
| alphaI_cluster_uncultured_forest_soil_bacterium | 1 | <0.01% |  | 2 | <0.01% | Too_rare |
| Comamonadaceae_unclassified | 0 | <0.01% |  | 2 | <0.01% | Too_rare |
| Novosphingobium_capsulatum | 0 | <0.01% |  | 2 | <0.01% | Too_rare |
| AKYG597_uncultured_bacterium | 0 | <0.01% |  | 2 | <0.01% | Too_rare |
| Gordonibacter_pamelaeae | 0 | <0.01% |  | 2 | <0.01% | Too_rare |
| Anaerolineaceae_unclassified | 0 | <0.01% |  | 2 | <0.01% | Too_rare |
| Comamonadaceae_uncultured_burkholderiaceae_bacterium | 0 | <0.01% |  | 2 | <0.01% | Too_rare |
| swine_effluent_bacterium_CHNDP38 | 0 | <0.01% |  | 2 | <0.01% | Too_rare |
| Rhodospirillales_unclassified | 0 | <0.01% |  | 2 | <0.01% | Too_rare |
| Streptococcus_unclassified | 0 | <0.01% |  | 2 | <0.01% | Too_rare |
| Streptomyces_unclassified | 0 | <0.01% |  | 2 | <0.01% | Too_rare |
| Anaerofustis_unclassified | 0 | <0.01% |  | 2 | <0.01% | Too_rare |
| Flexibacter_unclassified | 0 | <0.01% |  | 2 | <0.01% | Too_rare |
| Myxococcales_uncultured_bacterium | 0 | <0.01% |  | 2 | <0.01% | Too_rare |
| Pirellula_uncultured_bacterium | 0 | <0.01% |  | 2 | <0.01% | Too_rare |
| Sporichthyaceae_unclassified | 0 | <0.01% |  | 2 | <0.01% | Too_rare |
| Streptacidiphilus_unclassified | 0 | <0.01% |  | 2 | <0.01% | Too_rare |
| AT.s3.28_uncultured_bacterium | 0 | <0.01% |  | 2 | <0.01% | Too_rare |
| AT425.EubC11_terrestrial_group_uncultured_gemmatimonadetes_bacterium | 0 | <0.01% |  | 2 | <0.01% | Too_rare |
| Achromobacter_uncultured_achromobacter_sp. | 0 | <0.01% |  | 2 | <0.01% | Too_rare |
| Adhaeribacter_uncultured_soil_bacterium | 0 | <0.01% |  | 2 | <0.01% | Too_rare |
| Aerococcus_uncultured_bacterium | 0 | <0.01% |  | 2 | <0.01% | Too_rare |
| Aquicella_unclassified | 0 | <0.01% |  | 2 | <0.01% | Too_rare |
| Aquicella_uncultured_soil_bacterium | 0 | <0.01% |  | 2 | <0.01% | Too_rare |
| Aurantimonas_sp._AU22 | 0 | <0.01% |  | 2 | <0.01% | Too_rare |
| B79_uncultured_bacterium | 0 | <0.01% |  | 2 | <0.01% | Too_rare |
| Brevibacterium_unclassified | 0 | <0.01% |  | 2 | <0.01% | Too_rare |
| CCM11a_uncultured_bacterium | 0 | <0.01% |  | 2 | <0.01% | Too_rare |
| Caldilineaceae_unclassified | 0 | <0.01% |  | 2 | <0.01% | Too_rare |
| Capnocytophaga_sputigena | 0 | <0.01% |  | 2 | <0.01% | Too_rare |
| Catelliglobosispora_unclassified | 0 | <0.01% |  | 2 | <0.01% | Too_rare |
| Caulobacter_unclassified | 0 | <0.01% |  | 2 | <0.01% | Too_rare |
| DS.100_uncultured_bacterium | 0 | <0.01% |  | 2 | <0.01% | Too_rare |
| Deinococcus_uncultured_deinococcus_sp. | 0 | <0.01% |  | 2 | <0.01% | Too_rare |
| Flavobacterium_uncultured_bacterium | 0 | <0.01% |  | 2 | <0.01% | Too_rare |
| Gaiellales_uncultured_bacterium | 0 | <0.01% |  | 2 | <0.01% | Too_rare |
| Gaiellales_uncultured_rubrobacteridae_bacterium | 0 | <0.01% |  | 2 | <0.01% | Too_rare |
| Geothrix_uncultured_geothrix_sp. | 0 | <0.01% |  | 2 | <0.01% | Too_rare |
| Gitt.GS.136_uncultured_bacterium | 0 | <0.01% |  | 2 | <0.01% | Too_rare |
| Gluconobacter_albidus | 0 | <0.01% |  | 2 | <0.01% | Too_rare |
| KD2.123_uncultured_gemmatimonadetes_bacterium | 0 | <0.01% |  | 2 | <0.01% | Too_rare |
| LD12_freshwater_group_unclassified | 0 | <0.01% |  | 2 | <0.01% | Too_rare |
| Leptothrix_uncultured_organism | 0 | <0.01% |  | 2 | <0.01% | Too_rare |
| Neisseriaceae_uncultured_bacterium | 0 | <0.01% |  | 2 | <0.01% | Too_rare |
| Nitrosomonadaceae_uncultured_nitrosomonadales_bacterium | 0 | <0.01% |  | 2 | <0.01% | Too_rare |
| Nitrosospira_uncultured_beta_proteobacterium | 0 | <0.01% |  | 2 | <0.01% | Too_rare |
| Nitrospira_uncultured_bacterium | 0 | <0.01% |  | 2 | <0.01% | Too_rare |
| OPB56_unclassified | 0 | <0.01% |  | 2 | <0.01% | Too_rare |
| Pirellula_unclassified | 0 | <0.01% |  | 2 | <0.01% | Too_rare |
| Pla4_lineage_uncultured_bacterium | 0 | <0.01% |  | 2 | <0.01% | Too_rare |
| Planctomyces_unclassified | 0 | <0.01% |  | 2 | <0.01% | Too_rare |
| SHA.109_uncultured_bacterium | 0 | <0.01% |  | 2 | <0.01% | Too_rare |
| Shewanella_putrefaciens_200 | 0 | <0.01% |  | 2 | <0.01% | Too_rare |
| Streptococcus_sanguinis | 0 | <0.01% |  | 2 | <0.01% | Too_rare |
| Subgroup_22_unclassified | 0 | <0.01% |  | 2 | <0.01% | Too_rare |
| YNPFFP1_uncultured_bacterium | 0 | <0.01% |  | 2 | <0.01% | Too_rare |
| Alcaligenaceae_bacterium_BL.169 | 9 | 0.01% |  | 1 | <0.01% | Too_rare |
| Chryseobacterium_indologenes | 3 | <0.01% |  | 1 | <0.01% | Too_rare |
| Erythrobacteraceae_unclassified | 3 | <0.01% |  | 1 | <0.01% | Too_rare |
| Rhizobium_larrymoorei | 3 | <0.01% |  | 1 | <0.01% | Too_rare |
| Synechococcus_uncultured_bacterium | 3 | <0.01% |  | 1 | <0.01% | Too_rare |
| Anaerovorax_uncultured_bacterium | 2 | <0.01% |  | 1 | <0.01% | Too_rare |
| Armatimonadetes_uncultured_bacterium | 2 | <0.01% |  | 1 | <0.01% | Too_rare |
| Aerococcus_christensenii | 2 | <0.01% |  | 1 | <0.01% | Too_rare |
| Solirubrobacterales_unclassified | 2 | <0.01% |  | 1 | <0.01% | Too_rare |
| Ideonella_uncultured_bacterium | 1 | <0.01% |  | 1 | <0.01% | Too_rare |
| Nitrospira_uncultured_soil_bacterium | 1 | <0.01% |  | 1 | <0.01% | Too_rare |
| Arcobacter_unclassified | 1 | <0.01% |  | 1 | <0.01% | Too_rare |
| Microbacterium_sp._AC35 | 1 | <0.01% |  | 1 | <0.01% | Too_rare |
| Sphingomonas_echinoides | 1 | <0.01% |  | 1 | <0.01% | Too_rare |
| Gemmata_unclassified | 1 | <0.01% |  | 1 | <0.01% | Too_rare |
| Sphingomonas_paucimobilis | 1 | <0.01% |  | 1 | <0.01% | Too_rare |
| Bryobacter_uncultured_bacterium | 1 | <0.01% |  | 1 | <0.01% | Too_rare |
| Schlesneria_uncultured_bacterium | 1 | <0.01% |  | 1 | <0.01% | Too_rare |
| Clostridium_sporosphaeroides | 0 | <0.01% |  | 1 | <0.01% | Too_rare |
| Selenomonadales_unclassified | 0 | <0.01% |  | 1 | <0.01% | Too_rare |
| Bacteroides_graminisolvens | 0 | <0.01% |  | 1 | <0.01% | Too_rare |
| Epulopiscium_unclassified | 0 | <0.01% |  | 1 | <0.01% | Too_rare |
| Pyramidobacter_uncultured_synergistes_sp. | 0 | <0.01% |  | 1 | <0.01% | Too_rare |
| Exiguobacterium_sp._AT1b | 0 | <0.01% |  | 1 | <0.01% | Too_rare |
| Paracoccus_unclassified | 0 | <0.01% |  | 1 | <0.01% | Too_rare |
| Planobacterium_unclassified | 0 | <0.01% |  | 1 | <0.01% | Too_rare |
| Candidate_division_TM7_uncultured_bacterium | 0 | <0.01% |  | 1 | <0.01% | Too_rare |
| Flavobacterium_uncultured_flavobacterium_sp. | 0 | <0.01% |  | 1 | <0.01% | Too_rare |
| Nubsella_uncultured_bacterium | 0 | <0.01% |  | 1 | <0.01% | Too_rare |
| Solimonadaceae_uncultured_bacterium | 0 | <0.01% |  | 1 | <0.01% | Too_rare |
| Dechloromonas_uncultured_bacterium | 0 | <0.01% |  | 1 | <0.01% | Too_rare |
| Solimonadaceae_uncultured_sinobacteraceae_bacterium | 0 | <0.01% |  | 1 | <0.01% | Too_rare |
| Deinococcus_uncultured_bacterium | 0 | <0.01% |  | 1 | <0.01% | Too_rare |
| Nocardioides_unclassified | 0 | <0.01% |  | 1 | <0.01% | Too_rare |
| Rhizobiales_unclassified | 0 | <0.01% |  | 1 | <0.01% | Too_rare |
| Hydrogenophaga_unclassified | 0 | <0.01% |  | 1 | <0.01% | Too_rare |
| Nitrosomonadaceae_uncultured_delta_proteobacterium | 0 | <0.01% |  | 1 | <0.01% | Too_rare |
| Oxalophagus_uncultured_bacterium | 0 | <0.01% |  | 1 | <0.01% | Too_rare |
| Xanthomonadaceae_unclassified | 0 | <0.01% |  | 1 | <0.01% | Too_rare |
| vadinBC27_wastewater.sludge_group_unclassified | 0 | <0.01% |  | 1 | <0.01% | Too_rare |
| Aquabacterium_unclassified | 0 | <0.01% |  | 1 | <0.01% | Too_rare |
| Burkholderia_sp._IMP5GC | 0 | <0.01% |  | 1 | <0.01% | Too_rare |
| Byssovorax_uncultured_bacterium | 0 | <0.01% |  | 1 | <0.01% | Too_rare |
| Flavobacteriaceae_unclassified | 0 | <0.01% |  | 1 | <0.01% | Too_rare |
| Lapillicoccus_unclassified | 0 | <0.01% |  | 1 | <0.01% | Too_rare |
| Marmoricola_unclassified | 0 | <0.01% |  | 1 | <0.01% | Too_rare |
| Nitrobacter_uncultured_rhodopseudomonas_sp. | 0 | <0.01% |  | 1 | <0.01% | Too_rare |
| Phenylobacterium_uncultured_bacterium | 0 | <0.01% |  | 1 | <0.01% | Too_rare |
| Sphingobacterium_daejeonense | 0 | <0.01% |  | 1 | <0.01% | Too_rare |
| Thermomonas_uncultured_xanthomonadales_bacterium | 0 | <0.01% |  | 1 | <0.01% | Too_rare |
| Xanthobacteraceae_uncultured_hyphomicrobiaceae_bacterium | 0 | <0.01% |  | 1 | <0.01% | Too_rare |
| B1.7BS_uncultured_bacterium | 0 | <0.01% |  | 1 | <0.01% | Too_rare |
| Bacillus_uncultured_soil_bacterium | 0 | <0.01% |  | 1 | <0.01% | Too_rare |
| Blastocatella_uncultured_soil_bacterium | 0 | <0.01% |  | 1 | <0.01% | Too_rare |
| Candidatus_Solibacter_uncultured_acidobacteriaceae_bacterium | 0 | <0.01% |  | 1 | <0.01% | Too_rare |
| Chitinophagaceae_uncultured_bacterium | 0 | <0.01% |  | 1 | <0.01% | Too_rare |
| Chryseobacterium_uncultured_bacterium | 0 | <0.01% |  | 1 | <0.01% | Too_rare |
| Corynebacterium_sp._NML94.0264 | 0 | <0.01% |  | 1 | <0.01% | Too_rare |
| DS.100_uncultured_acidobacteria_bacterium | 0 | <0.01% |  | 1 | <0.01% | Too_rare |
| Iamia_unclassified | 0 | <0.01% |  | 1 | <0.01% | Too_rare |
| Janibacter_limosus | 0 | <0.01% |  | 1 | <0.01% | Too_rare |
| Myxococcales_unclassified | 0 | <0.01% |  | 1 | <0.01% | Too_rare |
| Nitrosomonadaceae_uncultured_uromyces | 0 | <0.01% |  | 1 | <0.01% | Too_rare |
| Nitrosomonas_uncultured_bacterium | 0 | <0.01% |  | 1 | <0.01% | Too_rare |
| OM190_uncultured_bacterium | 0 | <0.01% |  | 1 | <0.01% | Too_rare |
| OPB35_soil_group_uncultured_bacterium | 0 | <0.01% |  | 1 | <0.01% | Too_rare |
| Pedomicrobium_unclassified | 0 | <0.01% |  | 1 | <0.01% | Too_rare |
| Pedomicrobium_uncultured_bacterium | 0 | <0.01% |  | 1 | <0.01% | Too_rare |
| Pirellula_uncultured_soil_bacterium | 0 | <0.01% |  | 1 | <0.01% | Too_rare |
| Proteocatella_uncultured_bacterium | 0 | <0.01% |  | 1 | <0.01% | Too_rare |
| RB41_uncultured_bacterium | 0 | <0.01% |  | 1 | <0.01% | Too_rare |
| Streptococcus_gordonii | 0 | <0.01% |  | 1 | <0.01% | Too_rare |
| Subgroup_5_uncultured_acidobacterium_sp. | 0 | <0.01% |  | 1 | <0.01% | Too_rare |
| Subgroup_7_unclassified | 0 | <0.01% |  | 1 | <0.01% | Too_rare |
| Thermoanaerobacterium_uncultured_bacterium | 0 | <0.01% |  | 1 | <0.01% | Too_rare |
| X0319.6M6_uncultured_bacterium | 0 | <0.01% |  | 1 | <0.01% | Too_rare |
| A0839_unclassified | 0 | <0.01% |  | 1 | <0.01% | Too_rare |
| Actinobacteria_unclassified | 0 | <0.01% |  | 1 | <0.01% | Too_rare |
| Altererythrobacter_uncultured_bacterium | 0 | <0.01% |  | 1 | <0.01% | Too_rare |
| Anaeromyxobacter_unclassified | 0 | <0.01% |  | 1 | <0.01% | Too_rare |
| Arenimonas_uncultured_bacterium | 0 | <0.01% |  | 1 | <0.01% | Too_rare |
| B1.7BS_uncultured_soil_bacterium | 0 | <0.01% |  | 1 | <0.01% | Too_rare |
| BHI80.139_uncultured_geobacillus_sp. | 0 | <0.01% |  | 1 | <0.01% | Too_rare |
| Bryobacter_unclassified | 0 | <0.01% |  | 1 | <0.01% | Too_rare |
| Caenimonas_uncultured_bacterium | 0 | <0.01% |  | 1 | <0.01% | Too_rare |
| Candidate_division_OP3_uncultured_bacterium | 0 | <0.01% |  | 1 | <0.01% | Too_rare |
| Candidate_division_TM7_uncultured_soil_bacterium | 0 | <0.01% |  | 1 | <0.01% | Too_rare |
| Candidatus_Nitrospira_defluvii | 0 | <0.01% |  | 1 | <0.01% | Too_rare |
| DA111_unclassified | 0 | <0.01% |  | 1 | <0.01% | Too_rare |
| Elev.16S.1158_uncultured_bacterium | 0 | <0.01% |  | 1 | <0.01% | Too_rare |
| Elizabethkingia_meningoseptica | 0 | <0.01% |  | 1 | <0.01% | Too_rare |
| Flexibacter_uncultured_bacterium | 0 | <0.01% |  | 1 | <0.01% | Too_rare |
| GR.WP33.30_uncultured_desulfuromonas_sp. | 0 | <0.01% |  | 1 | <0.01% | Too_rare |
| GR.WP33.30_uncultured_soil_bacterium | 0 | <0.01% |  | 1 | <0.01% | Too_rare |
| Gemmatimonadaceae_uncultured_gemmatimonadales_bacterium | 0 | <0.01% |  | 1 | <0.01% | Too_rare |
| Gemmatimonadaceae_uncultured_gemmatimonadetes_bacterium | 0 | <0.01% |  | 1 | <0.01% | Too_rare |
| Gemmatimonas_unclassified | 0 | <0.01% |  | 1 | <0.01% | Too_rare |
| Gemmatimonas_uncultured_gemmatimonadales_bacterium | 0 | <0.01% |  | 1 | <0.01% | Too_rare |
| Gemmatimonas_uncultured_gemmatimonadetes_bacterium | 0 | <0.01% |  | 1 | <0.01% | Too_rare |
| Hirschia_uncultured_bacterium | 0 | <0.01% |  | 1 | <0.01% | Too_rare |
| I.10_uncultured_alpha_proteobacterium | 0 | <0.01% |  | 1 | <0.01% | Too_rare |
| Intrasporangiaceae_unclassified | 0 | <0.01% |  | 1 | <0.01% | Too_rare |
| KCM.B.60_uncultured_bacterium | 0 | <0.01% |  | 1 | <0.01% | Too_rare |
| Lactococcus_piscium | 0 | <0.01% |  | 1 | <0.01% | Too_rare |
| Lineage_IIb_uncultured_bacterium | 0 | <0.01% |  | 1 | <0.01% | Too_rare |
| Lysinibacillus_xylanilyticus | 0 | <0.01% |  | 1 | <0.01% | Too_rare |
| MNC12_uncultured_rhodospirillales_bacterium | 0 | <0.01% |  | 1 | <0.01% | Too_rare |
| Marmoricola_uncultured_bacterium | 0 | <0.01% |  | 1 | <0.01% | Too_rare |
| Methylocystis_unclassified | 0 | <0.01% |  | 1 | <0.01% | Too_rare |
| Microvirga_unclassified | 0 | <0.01% |  | 1 | <0.01% | Too_rare |
| Myxococcales_uncultured_cystobacteraceae_bacterium | 0 | <0.01% |  | 1 | <0.01% | Too_rare |
| Neisseria_unclassified | 0 | <0.01% |  | 1 | <0.01% | Too_rare |
| Paludibacter_uncultured_bacteroidetes_bacterium | 0 | <0.01% |  | 1 | <0.01% | Too_rare |
| Pir4_lineage_uncultured_bacterium | 0 | <0.01% |  | 1 | <0.01% | Too_rare |
| Pirellula_uncultured_pasteuria_sp. | 0 | <0.01% |  | 1 | <0.01% | Too_rare |
| Pirellula_uncultured_planctomycete | 0 | <0.01% |  | 1 | <0.01% | Too_rare |
| Pla4_lineage_uncultured_prokaryote | 0 | <0.01% |  | 1 | <0.01% | Too_rare |
| Planctomyces_uncultured_planctomyces_sp. | 0 | <0.01% |  | 1 | <0.01% | Too_rare |
| RB41_uncultured_acidobacteriaceae_bacterium | 0 | <0.01% |  | 1 | <0.01% | Too_rare |
| Rhodopirellula_uncultured_planctomycetaceae_bacterium | 0 | <0.01% |  | 1 | <0.01% | Too_rare |
| Rhodoplanes_unclassified | 0 | <0.01% |  | 1 | <0.01% | Too_rare |
| Rhodovibrio_uncultured_rhodospirillaceae_bacterium | 0 | <0.01% |  | 1 | <0.01% | Too_rare |
| SC.I.84_uncultured_beta_proteobacterium | 0 | <0.01% |  | 1 | <0.01% | Too_rare |
| SM1A02_unclassified | 0 | <0.01% |  | 1 | <0.01% | Too_rare |
| SM2F11_uncultured_bacterium | 0 | <0.01% |  | 1 | <0.01% | Too_rare |
| Solirubrobacter_uncultured_bacterium | 0 | <0.01% |  | 1 | <0.01% | Too_rare |
| Sorangium_uncultured_bacterium | 0 | <0.01% |  | 1 | <0.01% | Too_rare |
| Subgroup_17_unclassified | 0 | <0.01% |  | 1 | <0.01% | Too_rare |
| Subgroup_17_uncultured_soil_bacterium | 0 | <0.01% |  | 1 | <0.01% | Too_rare |
| Subgroup_22_uncultured_bacterium | 0 | <0.01% |  | 1 | <0.01% | Too_rare |
| TK34_uncultured_bacterium | 0 | <0.01% |  | 1 | <0.01% | Too_rare |
| TakashiAC.B11_uncultured_actinobacterium | 0 | <0.01% |  | 1 | <0.01% | Too_rare |
| Terrimonas_uncultured_bacterium | 0 | <0.01% |  | 1 | <0.01% | Too_rare |
| Thermomonas_uncultured_xanthomonadaceae_bacterium | 0 | <0.01% |  | 1 | <0.01% | Too_rare |
| Tissierella_unclassified | 0 | <0.01% |  | 1 | <0.01% | Too_rare |
| Virgisporangium_ochraceum | 0 | <0.01% |  | 1 | <0.01% | Too_rare |
| WCHB1.32_uncultured_bacterium | 0 | <0.01% |  | 1 | <0.01% | Too_rare |
| Xanthobacter_unclassified | 0 | <0.01% |  | 1 | <0.01% | Too_rare |
| Xanthobacteraceae_uncultured_rhodocyclaceae_bacterium | 0 | <0.01% |  | 1 | <0.01% | Too_rare |
| hgcI_clade_uncultured_candidatus_planktophila_sp. | 0 | <0.01% |  | 1 | <0.01% | Too_rare |
| planctomycete_A.2 | 0 | <0.01% |  | 1 | <0.01% | Too_rare |
| Roseburia_unclassified | 14 | 0.02% |  | 0 | <0.01% | Too_rare |
| Catabacter_hongkongensis | 14 | 0.02% |  | 0 | <0.01% | Too_rare |
| Christensenella_uncultured_clostridium_sp. | 12 | 0.02% |  | 0 | <0.01% | Too_rare |
| Parabacteroides_uncultured_parabacteroides_sp. | 7 | 0.01% |  | 0 | <0.01% | Too_rare |
| Akkermansia_uncultured_verrucomicrobiaceae_bacterium | 5 | 0.01% |  | 0 | <0.01% | Too_rare |
| Clostridium_sp._strain_S6 | 5 | 0.01% |  | 0 | <0.01% | Too_rare |
| Singulisphaera_uncultured_soil_bacterium | 5 | 0.01% |  | 0 | <0.01% | Too_rare |
| Eubacterium_sp._Pei061 | 4 | 0.01% |  | 0 | <0.01% | Too_rare |
| Ruminococcus_unclassified | 4 | 0.01% |  | 0 | <0.01% | Too_rare |
| unidentified_rumen_bacterium_12.124 | 4 | 0.01% |  | 0 | <0.01% | Too_rare |
| Clostridium_ramosum | 3 | <0.01% |  | 0 | <0.01% | Too_rare |
| Christensenella_uncultured_bacterium | 3 | <0.01% |  | 0 | <0.01% | Too_rare |
| Lactobacillus_salivarius | 3 | <0.01% |  | 0 | <0.01% | Too_rare |
| Bacillus_nealsonii | 2 | <0.01% |  | 0 | <0.01% | Too_rare |
| Eubacterium_unclassified | 2 | <0.01% |  | 0 | <0.01% | Too_rare |
| Blastococcus_unclassified | 2 | <0.01% |  | 0 | <0.01% | Too_rare |
| Chroococcidiopsis_unclassified | 2 | <0.01% |  | 0 | <0.01% | Too_rare |
| JG35.K1.AG5_uncultured_bacterium | 2 | <0.01% |  | 0 | <0.01% | Too_rare |
| Limnobacter_unclassified | 2 | <0.01% |  | 0 | <0.01% | Too_rare |
| RsaHF231_uncultured_bacterium | 2 | <0.01% |  | 0 | <0.01% | Too_rare |
| SM1A07_unclassified | 2 | <0.01% |  | 0 | <0.01% | Too_rare |
| Lactobacillus_plantarum | 1 | <0.01% |  | 0 | <0.01% | Too_rare |
| Clostridium_sp._Culture_Jar.13 | 1 | <0.01% |  | 0 | <0.01% | Too_rare |
| Candidatus_Koribacter_uncultured_bacterium | 1 | <0.01% |  | 0 | <0.01% | Too_rare |
| Curtobacterium_luteum | 1 | <0.01% |  | 0 | <0.01% | Too_rare |
| Variovorax_unclassified | 1 | <0.01% |  | 0 | <0.01% | Too_rare |
| X480.2_uncultured_rubrobacteridae_bacterium | 1 | <0.01% |  | 0 | <0.01% | Too_rare |
| Acetobacteraceae_unclassified | 1 | <0.01% |  | 0 | <0.01% | Too_rare |
| Acidimicrobiales_uncultured_aciditerrimonas_sp. | 1 | <0.01% |  | 0 | <0.01% | Too_rare |
| Acidimicrobiales_uncultured_bacterium | 1 | <0.01% |  | 0 | <0.01% | Too_rare |
| Chitinophagaceae_uncultured_prokaryote | 1 | <0.01% |  | 0 | <0.01% | Too_rare |
| Chloroplast_uncultured_soil_bacterium | 1 | <0.01% |  | 0 | <0.01% | Too_rare |
| Cyanobacteria_uncultured_bacterium | 1 | <0.01% |  | 0 | <0.01% | Too_rare |
| Deinococcus_uncultured_organism | 1 | <0.01% |  | 0 | <0.01% | Too_rare |
| Helicobacter_pylori | 1 | <0.01% |  | 0 | <0.01% | Too_rare |
| MNG7_uncultured_bacterium | 1 | <0.01% |  | 0 | <0.01% | Too_rare |
| Patulibacter_unclassified | 1 | <0.01% |  | 0 | <0.01% | Too_rare |
| TRA3.20_uncultured_soil_bacterium | 1 | <0.01% |  | 0 | <0.01% | Too_rare |
| Terrimonas_unclassified | 1 | <0.01% |  | 0 | <0.01% | Too_rare |
| Xanthomonadales_uncultured_eubacterium_wd2124 | 1 | <0.01% |  | 0 | <0.01% | Too_rare |
