# Supplementary figures and images for: Changes of diet and dominant intestinal microbes in farmland frogs
Source: BMC Microbiol. 2016 Mar 10;16:33. doi: 10.1186/s12866-016-0660-4 (PMC4785643; doi:10.1186/s12866-016-0660-4)

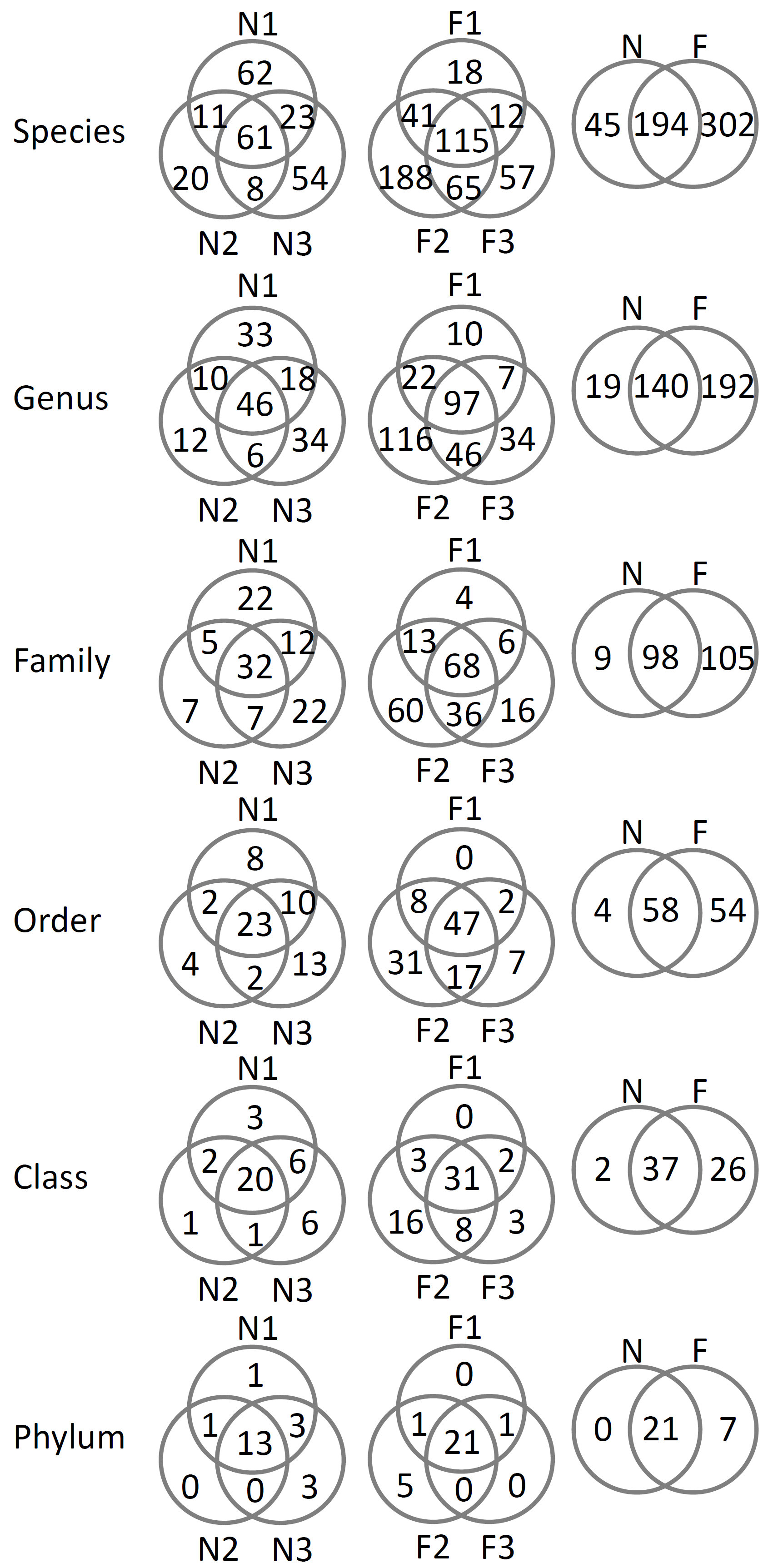

Supplement: Additional file 2: Figure S1. — Venn diagram representing the species diversity of the intestinal microbial taxa in frog intestines. The numbers in circles represent the number of taxa. As can be seen, a higher number of unique microbial taxa were found in farmland frogs (for which the individual F2 made the highest contribution). (TIF 17137 kb) [file 12866_2016_660_MOESM2_ESM.tif]

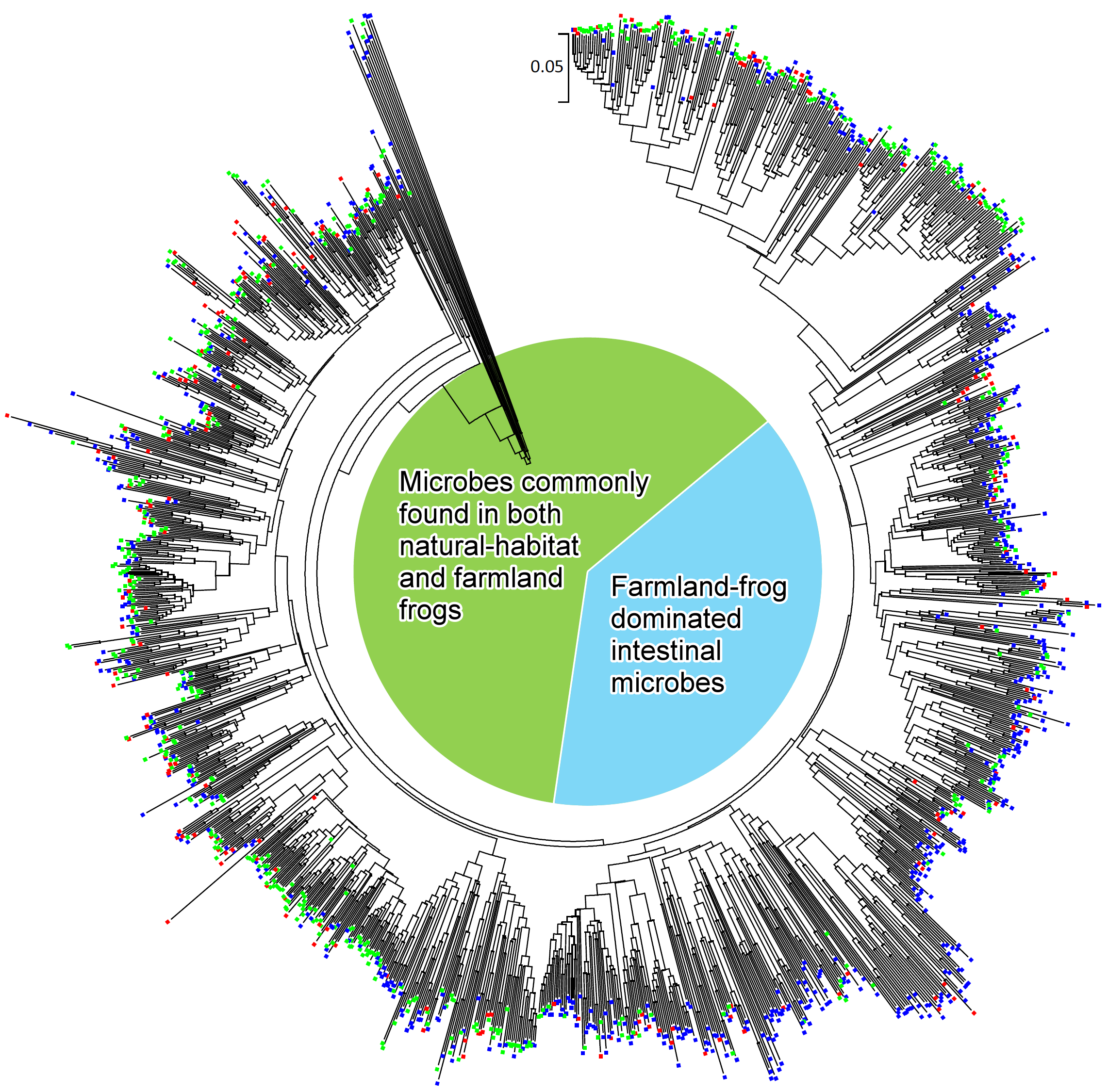

Supplement: Additional file 3: Figure S2. — The Neighbor-joining tree reveals the phylogenetic position of the intestinal microbes of Fejervarya limnocharis in farmland only (blue), in natural habitats only (red), and in both environments (green). The phylogenetic analysis showed that roughly 1/3 clades of microbial species are found in the farmland frogs only. (TIF 15121 kb) [file 12866_2016_660_MOESM3_ESM.tif]

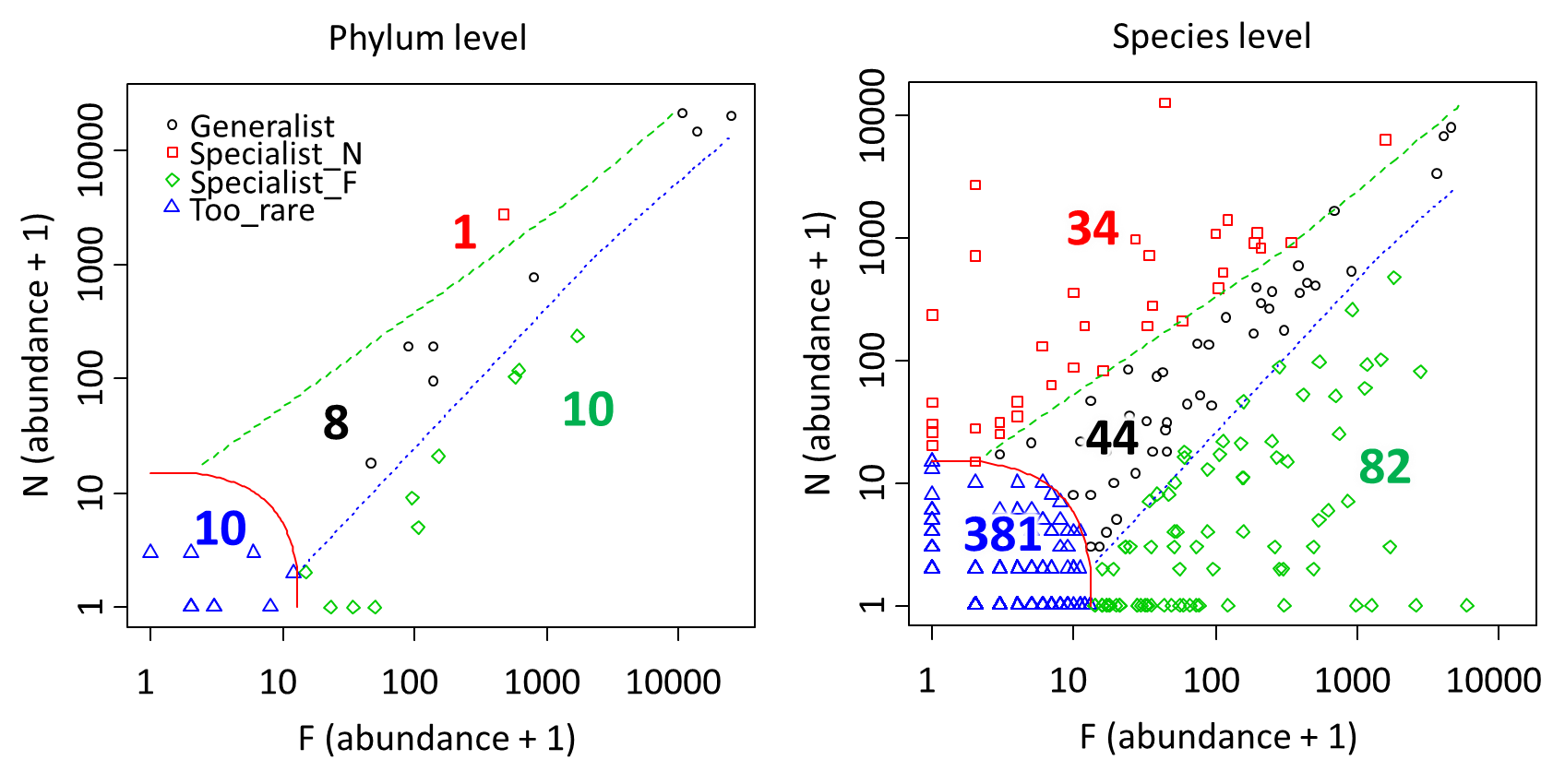

Supplement: Additional file 4: Figure S3. — Categories of gut bacteria between habitats classified by multinomial species classification method (CLAM). Classification in phylum level and species level are shown. Values in plots indicate the number of bacterial taxa (i.e. phyla and species) of each category. F and N in the x- and y-axes indicate the farmland and natural habitat, respectively. (TIF 156 kb) [file 12866_2016_660_MOESM4_ESM.tif]

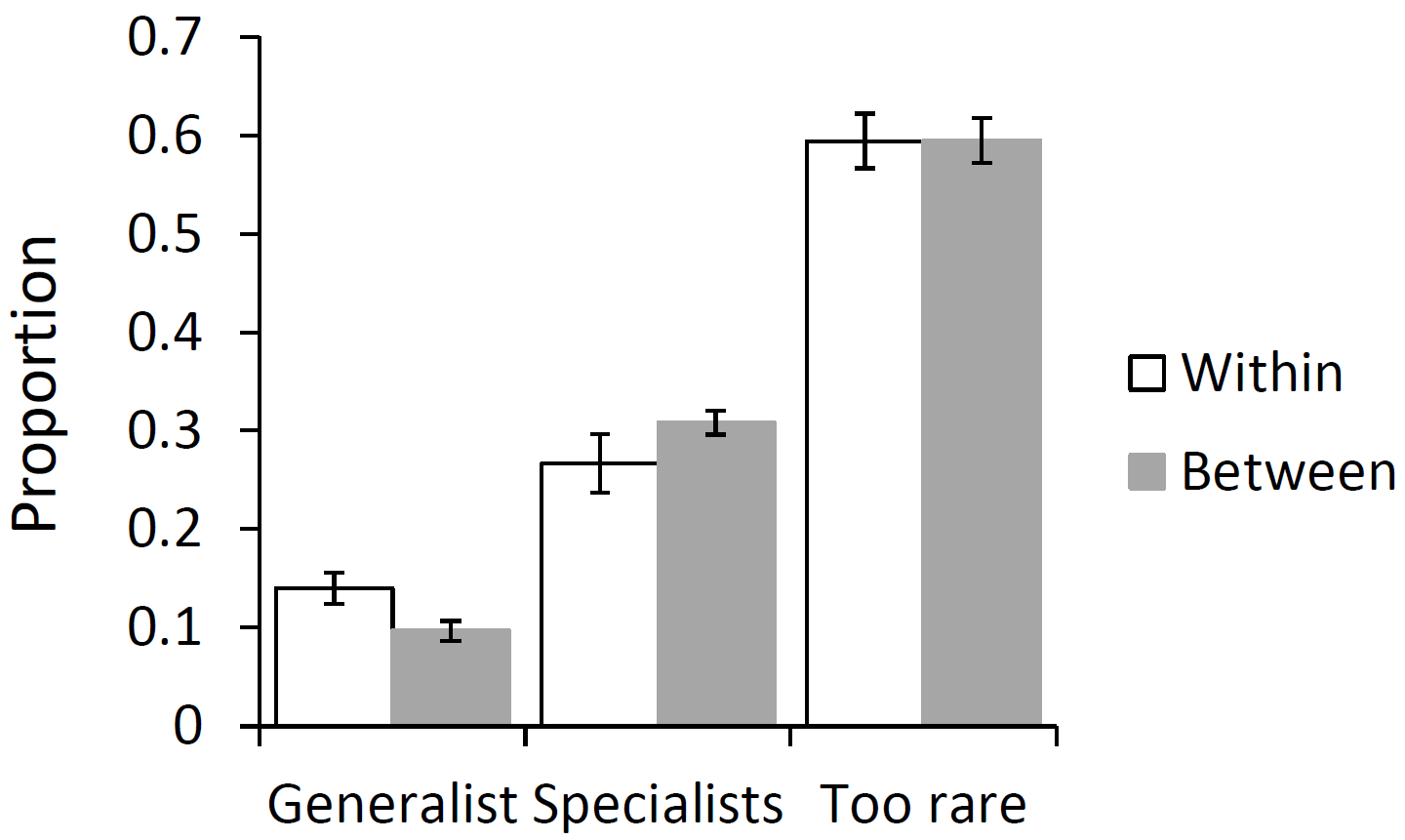

Supplement: Additional file 5: Figure S4. — Pairwise comparison of gut bacterial categories of individual hosts within habitats and between habitats. All three comparisons of generalists, specialists, and too-rare type of bacterial communities within habitats and between habitats are non-significant different. (TIF 3668 kb) [file 12866_2016_660_MOESM5_ESM.tif]
